# Rhenium Tricarbonyl Complexes of Azodicarboxylate Ligands

**DOI:** 10.3390/molecules27238159

**Published:** 2022-11-23

**Authors:** Rose Jordan, Maryam Niazi, Sascha Schäfer, Wolfgang Kaim, Axel Klein

**Affiliations:** 1 Department für Chemie, Institut für Anorganische Chemie, Mathematisch-Naturwissenschaftliche Fakultät, Universität zu Köln, Greinstrasse 6, D-50939 Köln, Germany; 2 Institut für Anorganische Chemie, Universität Stuttgart, Pfaffenwaldring 55, D-70550 Stuttgart, Germany

**Keywords:** carbonylrhenium(I), azodicarboxylate ligand, NIR absorption, electrochemistry, DFT

## Abstract

The excellent π-accepting azodicarboxylic esters adcOR (R = Et, *i*Pr, *t*Bu, Bn (CH_2_-C_6_H_5_) and Ph) and the piperidinyl amide derivative adcpip were used as bridging chelate ligands in dinuclear Re(CO)_3_ complexes [{Re(CO)_3_Cl}_2_(µ-adcOR)] and [{Re(CO)_3_Cl}_2_(µ-adcpip)]. From the adcpip ligand the mononuclear derivatives [Re(CO)_3_Cl(adcpip)] and [Re(CO)_3_(PPh_3_)(µ-adcpip)]Cl were also obtained. Optimised geometries from density functional theory (DFT) calculations show *syn* and *anti* isomers for the dinuclear *fac*-Re(CO)_3_ complexes at slightly different energies but they were not distinguishable from experimental IR or UV–Vis absorption spectroscopy. The electrochemistry of the adc complexes showed reduction potentials slightly below 0.0 V vs. the ferrocene/ferrocenium couple. Attempts to generate the radicals [{Re(CO)_3_Cl}_2_(µ-adcOR)]^•−^ failed as they are inherently unstable, losing very probably first the Cl^−^ coligand and then rapidly cleaving one [Re(CO)_3_] fragment. Consequently, we found signals in EPR very probably due to mononuclear radical complexes [Re(CO)_3_(solv)(adc)]^•^. The underlying Cl^−^→solvent exchange was modelled for the mononuclear [Re(CO)_3_Cl(adcpip)] using DFT calculations and showed a markedly enhanced Re-Cl labilisation for the reduced compared with the neutral complex. Both the easy reduction with potentials ranging roughly from −0.2 to −0.1 V for the adc ligands and the low-energy NIR absorptions in the 700 to 850 nm range place the adc ligands with their lowest-lying π* orbital being localised on the azo function, amongst comparable bridging chelate N^N coordinating ligands with low-lying π* orbitals of central azo, tetrazine or pyrazine functions. Comparative (TD)DFT-calculations on the Re(CO)_3_Cl complexes of the adcpip ligand using the quite established basis set and functionals M06-2X/def2TZVP/LANL2DZ/CPCM(THF) and the more advanced TPSSh/def2-TZVP(+def2-ECP for Re)/CPCMC(THF) for single-point calculations with BP86/def2-TZVP(+def2-ECP for Re)/CPCMC(THF) optimised geometries showed a markedly better agreement of the latter with the experimental XRD, IR and UV–Vis absorption data.

## 1. Introduction

**Scheme 1:**
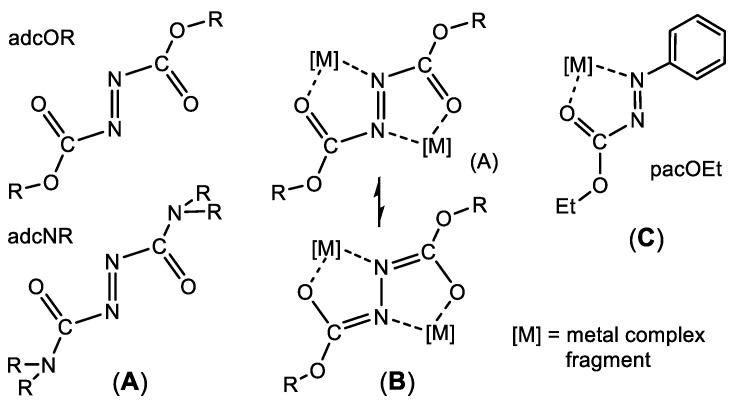
Azo dicarboxylate derivatives (esters = adcOR, amides = adcNR, left) (**A**), their double metal coordination enforcing electron delocalisation in the OCNNCO unit (**B**), and the monodentate phenylcarboxylic ethyl ester ligand (pacOEt) (**C**).

**Scheme 2:**
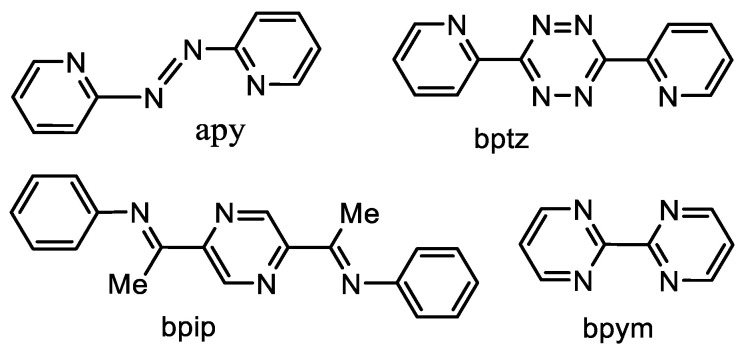
µ-κ^2^,κ^2^-*N*,*N*-bridging ligands with low-lying π* orbitals.

The main application of azo dicarboxylic esters or amides (adcOR or adcNR, Scheme 1) lies in their use as catalysts in the versatile Mitsunobu reaction which allows conversion of alcohols into a variety of functional groups, such as esters [1,2,3,4], and in a number of further organic reactions in which they act as (co)catalyst [5,6,7,8,9,10], or were used as reactive building blocks [11,12,13,14]. At the same time, they are also interesting ligands forming one or two five-ring N^O chelates (Scheme 1) [15,16,17,18,19,20,21,22,23,24,25,26,27,28,29,30,31,32,33,34,35,36,37,38,39,40,41,42]. In dinuclear complexes, these coplanar edge-sharing five-membered chelate rings allow very close M … M contacts (<500 pm). Their very low-lying π* orbitals are not only responsible for intense and low-energy metal-to-ligand charge transfer bands in corresponding complexes [17,18,22,23,25,26,27,28,29,32,34,35,36,37] but also for very high reduction potentials of such complexes often around 0 V (vs. NHE), allowing the facile preparation of radical complexes [22,23,26,27,29,32,33,34,35,36,41] and in total they are able to store up to two electrons [22,26,27,29,32,33,34,35,36]. In this way, the adc ligands are prototypical redox-active/non-innocent ligands [15,21,23,29] and can be viewed together with further bridging µ-κ^2^,κ^2^ chelate ligands with very low-lying π* orbitals such as apy = 2,2′-azobipyridine; bptz = 2,5-bis(2-pyridyl)-1,3,4,6-tetrazine, bpip = 2,5-bis(1-phenyliminoethyl)pyrazine and bpym = 2,2′-bipyrimidine (Scheme 2) [43,44,45,46,47,48,49,50,51,52,53]. Dinuclear complexes of such ligands have been studied as low-energy electron transfer or low-energy absorbing materials for potential multi-electron catalysis or optoelectronic applications [21,27,28,29]. Moreover, the R groups of the esters or amides can be easily introduced and allow various steric and electronic tunings of the complexes [32,33,34,35]. Very recently, azo dicarboxylate ligands have also been used to synthesise transition metal complexes with cytotoxic properties [17,18].

**Scheme 3:**
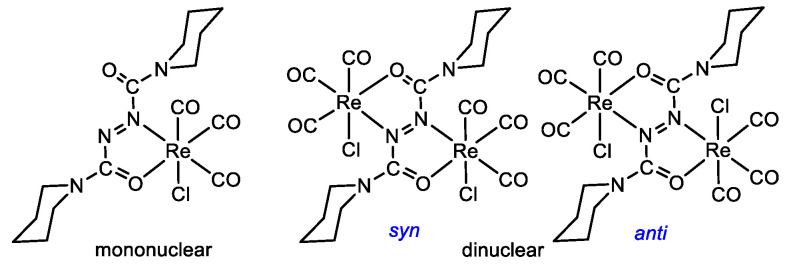
Mono- and dinuclear structures for Re(CO)_3_Cl complexes for the adcpip ligand.

Herein, we report on the reactions of five azo dicarboxylate ester ligands adcOR (R = Et, *i*Pr, *t*Bu, Ph, Bn, Scheme 1) and an amide derivative (adcpip, Scheme 3) with [Re(CO)_5_Cl] that we carried out in order to obtain mono- and dinuclear Re(CO)_3_Cl complexes (Scheme 3). For the sake of comparison, we also synthesized the phenylazocarboxylic ethyl ester (pacOEt) [54] model ligand which exclusively forms mononuclear complexes (Scheme 1, right). We geometry-optimised the structures using density functional theory (DFT). The fit with the experimental XRD and IR data was used for benchmarking of functionals and basis sets.

We also studied the electrochemical properties, EPR spectroscopy and UV–Vis–NIR absorption spectroscopy and combined the experiments with DFT and TD-DFT calculations to probe for the electronic properties of the complexes. Comparison will be drawn between the Re(CO)_3_Cl complexes of these adc ligands with those of the similar bridging µ-κ^2^,κ^2^ chelate ligands with very low-lying π* orbitals apy, bptz, bpip and bpym (Scheme 2) [43,44,45,46,47,48,49,50,51,52,53].

## 2. Results and Discussion

### 2.1. Syntheses

When stirring the adc ligands with [Re(CO)_5_Cl] in toluene/CH_2_Cl_2_ mixtures at ambient *T*, no reaction occurred. At higher *T*, starting from 60 °C, the reaction mixture turned reddish, indicating the formation of complexes. For the dinuclear complexes, the reaction mixtures then gradually turned dark and after a few hours dark-coloured reaction mixtures were obtained. After evaporation, the resulting dark materials were recrystallised from CH_2_Cl_2_/*n*-hexane and the resulting material extracted with toluene (for details, see Section 4).

**Scheme 4:**
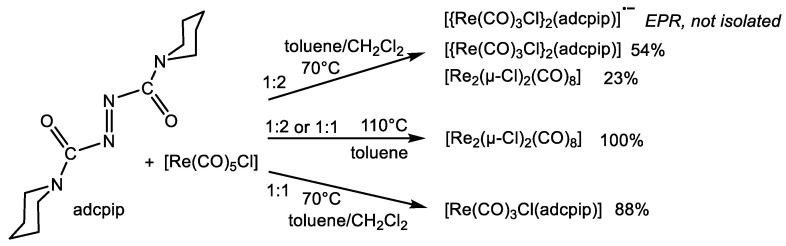
Reaction scheme for the reactions of adcpip with [Re(CO)_5_Cl].

This procedure allowed us to obtain dark green to black dinuclear complexes [{Re(CO)_3_Cl}_2_(µ-L)] from reactions using 1:2 (ligand:Re) ratios for the ligands containing low yields for R = Et (20%) and *i*Pr (26%) complexes and a moderate yield of 54% for the pip derivative (Scheme 4). For R = *t*Bu, Ph, Bn, the dark materials could not be analysed satisfactorily. The residual material from these reactions contained large amounts of the previously reported dinuclear complex [Re_2_(µ-Cl)_2_(CO)_8_] [55,56,57,58] and non-defined organic material, presumably from thermal decompositions of the adc ligands. The formation of [Re_2_(µ-Cl)_2_(CO)_8_] is not a dead-end as this dinuclear complex is also a reasonable Re(CO)_3_Cl precursor [55,56]. However, as [Re(CO)_5_Cl], it requires thermal activation.

From this, we conclude that the adc-OR ligands with the bulky *t*Bu, Ph and Bn substituents coordinated to slow due to their steric strain and decomposition of the ligands and their complexes is fast under the thermal activation conditions. For R = Et and *i*Pr and even more so the adcpip ligand, the complex formation is fast enough to compete with the decomposition. This means that this thermal synthesis method is on a razor’s edge between the necessary thermal activation of the Re precursors and the thermal decomposition of the azo-dicarboxylates. We also tried microwave and sonochemical activation, but the results were similar in that sizeable amounts of [Re_2_(µ-Cl)_2_(CO)_8_] were formed and yields of the complexes remained low.

When using 1:1 ligand to Re ratios, for R = Et or *i*Pr the dinuclear complexes [{Re(CO)_3_Cl}_2_(µ-L)] were also obtained. In contrast to this, for the adcpip derivative, the mononuclear dark violet complex [Re(CO)_3_Cl(adcpip)] was obtained in 88% yield from such a reaction solution. Under the same condition, we also obtained the dark red [Re(CO)_3_Cl(pacOEt)] (Scheme 1) in 75% yield.

The reactions were monitored by IR spectroscopy, showing the typical pattern (Supplementary Figure S1) for [Re(CO)_3_] fragments in their *facial* configuration [47,49,50,51,53,59,60,61,62,63]. IR also allowed the discrimination between mono- and dinuclear reaction products.

Based on previous experiences with the similar complex [{Re(CO)_3_Cl}_2_(µ-apy)] [47], we recorded EPR spectra during the reaction of [Re(CO)_5_Cl] and adcpip (2:1) and observed an 11-line EPR pattern in keeping with two Re centres of nuclear spin *I* = 5/2 (^185,187^Re; Supplementary Figure S2) in the assumed radical complex [{Re(CO)_3_Cl}_2_(adcpip)]^•−^. The observation of these radicals already in the reaction mixtures is in agreement with the very low-lying π* LUMOs of the ligand [22,23,25,26,27,28,29] allowing reduction during the synthesis procedure (although the reductant is not clear). However, this seems to occur only to a small part of the material as we obtained the binuclear complexes in their neutral, diamagnetic form after recrystallisation of the reaction product.

### 2.2. Exchange Reactions for the Chlorido Coligand

**Scheme 5:**
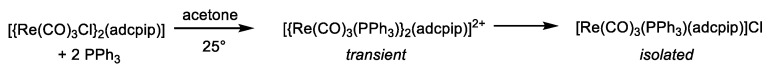
Reaction of [{Re(CO)_3_Cl}_2_(µ-adcpip)] with PPh_3_.

When trying to exchange the chlorido coligands in [{Re(CO)_3_Cl}_2_(µ-adcpip)] through PPh_3_, we recorded an IR spectrum after a few minutes’ reaction time, that clearly showed the three resonances for the [Re(CO)_3_] fragment markedly shifted to lower energy and two bands in the range 1700 to 1800 cm^−1^, indicating one coordinated and one uncoordinated C=O function of the adcpip ligand (Supplementary Figure S3A). The EPR spectrum of the reaction solution did not show the 11-line pattern typical for a dinuclear Re complex but a 9-line pattern representing presumably the mononuclear [Re(CO)_3_(PPh_3_)(adcpip)]^•^ radical complex (Supplementary Figure S3B). From this solution, we isolated [Re(CO)_3_(PPh_3_)(adcpip)]Cl through crystallisation. Thus, the dinuclear Re complex [{Re(CO)_3_Cl}_2_(µ-adcpip)] is very substitution labile and the resulting dinuclear PPh_3_ complex is not stable and decomposed to a mononuclear species (Scheme 5). The same formation of a mononuclear complex was observed when adding MeCN to a solution of [{Re(CO)_3_Cl}_2_(µ-adcpip)] (Supplementary Figure S4). Thus, the obtained dinuclear complexes can only be handled in non-coordinating solvent. At the same time, they are only sparsely soluble in aromatic solvent and not soluble in aliphatic hydrocarbons.

The IR spectra of the mononuclear and the dinuclear complexes are markedly different (Table 1 and Table S4 and Figures S1 and S7). The expected three CO stretching vibrations for the mononuclear [Re(CO)_3_Cl(adcpip)] are markedly higher in energy than those for the dinuclear [{Re(CO)_3_Cl}_2_(µ-adcpip)]. This shift is in keeping with the enhanced π backbonding in the dinuclear complex [27,29,32,44,53]. For the mononuclear [Re(CO)_3_Cl(adcpip)], a further band at 1704 cm^−1^ represents the uncoordinated adcpip CO group. Generally, the IR spectra of the Re(CO)_3_Cl complexes agree with those of previously reported *fac*-[Re(CO)_3_X] (X = any ligand) complexes showing three CO stretching resonances [47,49,50,51,52,53,59,60,61,62,63], the two at lower energy are sometimes merged into one broad band (Table 1).

When comparing different bridging ligands in dinuclear Re(CO)_3_Cl complexes, the CO stretching energies of the adc complexes do not differ markedly from those of related N^N bridging ligands, only the merging of bands II and III is more pronounced (Table 1).

Within our study, we made comparative DFT calculations on the Re(CO)_3_Cl complexes of the adcpip ligand using the quite established basis set and functionals M06-2X/def2TZVP/LANL2DZ/CPCM(THF), and the more advanced TPSSh/def2-TZVP(+def2-ECP for Re)/CPCMC(THF) for single-point calculations with BP86/def2-TZVP(+def2-ECP for Re)/CPCMC(THF) optimised geometries (see later, Table 2 and Table S2) and IR spectra (Figures S8 and S9, Table 1, Tables S3 and S4). The latter method gave markedly better agreement of calculated data with experimentally observed spectra. The CO stretching bands of the mononuclear [Re(CO)_3_Cl(adcpip)] ([Re]) complex are predicted at slightly lower energies than observed experimentally, with a systematic shift to lower wave numbers by about 30 cm^−1^. In contrast, the M06-2X/def2TZVP/LANL2DZ/CPCM(THF) method predicts vastly higher energies above 2100 cm^−1^ for these vibrations. For the dinuclear complexes ([Re]_2_), the situation is less simple, as a higher number of distinct vibrational modes were predicted, especially in the lower wavenumber range around 1900 cm^−1^ where one merged band is observed experimentally. While the assignment of individual predicted vibrations to the observed maxima is difficult, a slight redshift of all predicted modes compared to the experimental data is observed. Importantly, although we are sure that the sample contains both *syn* and *anti* isomers of [Re]_2_, the IR spectra gave no evidence for two species.

No NMR data of the binuclear complexes and [Re(CO)_3_Cl(pacOEt)] were obtained due to paramagnetic species impairing the measurements. From the mononuclear [Re(CO)_3_Cl(adcpip)], ^1^H NMR spectra showed the protons in the 1,5 positions of the piperidyl groups split into six components in agreement with the nuclear spin of ^185,187^Re of 5/2 (Figure S5). The ^13^C spectrum confirms the two non-equivalent piperidine groups (Figure S6).

### 2.3. Structures from X-ray Diffraction and DFT Calculations

**Figure 1:**
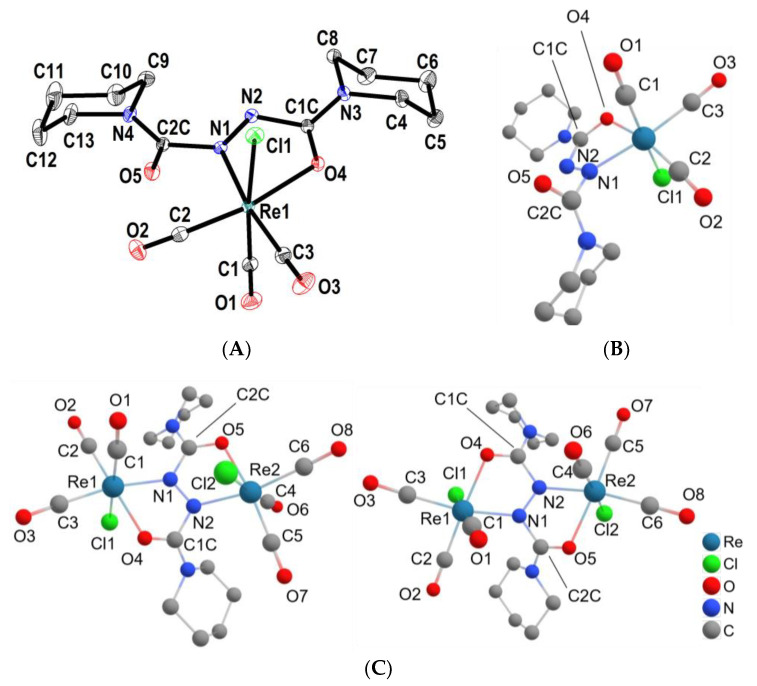
Experimental molecular structure of [Re(CO)_3_Cl(adcpip)] from single crystal X-ray diffraction (**A**), DFT-optimised structures in the *S_0_* ground state for the mononuclear [Re(CO)_3_Cl(adcpip)] ([Re]) (**B**) and dinuclear [{Re(CO)_3_Cl}_2_(µ-adcpip)] in *anti* (*anti*-[Re]_2_) and *syn* (*syn*-[Re]_2_) configuration (**C**); H atoms omitted for clarity; BP86/def2-TZVP(+def2-ECP for Re)/CPCMC(THF) level of theory.

By diffusion of *n*-heptane into a dilute solution of the mononuclear complex [Re(CO)_3_Cl(adcpip)] in EtOAc, we obtained single crystals of suitable quality and were able to solve the crystal structure in the monoclinic space group *P* 2_1_/*n* (see Figure 1A, crystal data in Table S1, metrics in Table 2 and Table S2). In contrast to this, crystallisation of the dinuclear adcpip compound failed.

Thus, we embarked on quantum chemical calculations using density functional theory (DFT) to model the dinuclear structures for the adcpip derivatives using two different methods as mentioned above. The experimental molecular structure parameters of [Re(CO)_3_Cl(adcpip)] (Table 2), the IR data (Table 1) as well as the UV/Vis absorption spectra (to be presented later) allowed unequivocal benchmarking in favour of BP86 optimised geometries and frequency calculations including TPSSh single-point property calculations and triple zeta basis sets for all elements (Figure 1). We thus present in the following only the results from this type of calculation. The key data from both the calculations using BP86/def2-TZVP(+def2-ECP for Re)/CPCMC(THF) or TPSSh/def2-TZVP(+def2-ECP for Re)/CPCMC(THF), and M06-2X/def2TZVP/LANL2DZ/CPCM(THF) are listed in the Supplementary Table S3.

Both the mononuclear [Re(CO)_3_Cl(adcpip)] and the dinuclear [{Re(CO)_3_Cl}_2_(µ-adcpip)] complex show the expected facial (*fac*) configuration of the three CO ligands (Figure 1). The geometry of the dinuclear [{Re(CO)_3_Cl}_2_(µ-adcpip)] is quite unsymmetric in both *anti* and *syn* forms. No centre of inversion was found for the *anti* isomer and the Cl–Re ... Re–Cl dihedral angles were 157° (*anti*) and 42° (*syn*), respectively. The calculated total energies say that the *syn* isomer is slightly more stable by 0.2 eV. The Re ... Re distance was calculated at 4.81 Å (*anti*) and 4.77 Å (*syn*), respectively.

The essential metrics of the [Re(CO)_3_Cl(N^O)] entities around the Re centres (Table S3A) were very similar to reported [Re(CO)_3_X(N^N)] structures with the exception that the N^O chelate bite with about 72° is markedly smaller than N^N chelate bites with values ranging from 80 to 83° [43,44,47,49,61,62,63,64,65].

### 2.4. Electrochemistry, EPR and DFT-Calculated Frontier Orbitals

**Figure 2:**
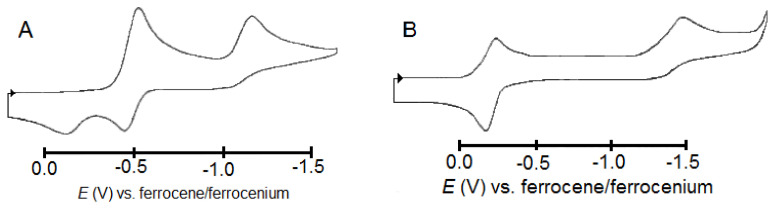
Cyclic voltammograms of [Re(CO)_3_Cl(adcpip)] (**A**) and [{Re(CO)_3_Cl}_2_(µ-adcpip)] (**B**) in 0.1 M *n-*Bu_4_NPF_6_/DCE.

**Electrochemistry:** Both mononuclear and dinuclear complexes show a first one-electron reversible reduction and a second irreversible reduction (Figure 2, data in Table 3). The irreversible character has been identified as being due to rapid cleavage of chloride after electrochemical reduction in comparable [Re(CO)_3_Cl(N^N)] (N^N = diimines) derivatives [46,47,48,49,50,59,61,64,65,66,67,68,69]. The dinuclear complexes exhibit very high reduction potential, for the adcpip complexes the potential of the mononuclear species lies lower by 0.3 V. For the uncoordinated adc ligands, we found two reduction processes at around −1 V and −1.9 V vs. the ferrocene/ferrocenium couple (Table S5). Assuming ligand-centred processes for these reductions, the coordination of Re(CO)_3_Cl causes massive anionic shifts, e.g., of more than 1 V upon coordination of one Re fragment to adcpip and more than 1.3 V when two Re fragments are coordinated.

For the complex [{Re(CO)_3_Cl}_2_(µ-adcOEt)], we found evidence for a split-wave for the first reduction (Figure S12) that we attributed to the *syn* and *anti* isomers based on the very similar behaviour of the complex [{Re(CO)_3_Cl}_2_(µ-apy)] (Table 3). For the other derivatives, we did not observe a splitting of the reduction waves. Interestingly, this phenomenon was first overseen for the Cl complex of the apy ligand [53], but was later found very pronounced for the F derivative and essentially absent for the Br and I congeners [44].

Irreversible oxidation waves were observed for all complexes slightly above 1.1 V and the dimeric [Re_2_(µ-Cl)_2_(CO)_8_] was oxidised reversibly at 1.32 V by two electrons (Figure S16).

When comparing the complex [{Re(CO)_3_Cl}_2_(µ-adcOEt)] with other dinuclear complexes of this ligand with the d^6^ systems [Ru(bpy)_2_]^4+^ (0.40 V) [32,36], and [Os(bpy)_2_]^4+^ (0.08 V) [32], and the d^10^ system [Cu(PPh_3_)_2_]^2+^ (0.42 V) [35], the Re(I) system has the lowest reduction potential which is probably due to the zero charge of the [Re(CO)_3_Cl] fragment. When comparing dinuclear Re(CO)_3_Cl complexes of different bringing ligands with very low-lying π* orbitals, the ease of reduction increases along the series bpip < adcpip < bptz < adcOiPr < adcOEt < bpym < apy places the adc ligands between the pyrazine-pyridine acceptor of bpip (bpip = 2,5-bis(1-phenyliminoethyl)pyrazine) [50] and the azo-pyridine ligand apy (2,2′-azobipyridine) [49,52,53].

**EPR spectroscopy:** A detailed EPR study of the radical anions [{Re(CO)_3_Cl}_2_(µ-adc)]^•−^ was prevented by their inherent instability. Attempts to reduce the dinuclear complexes [{Re(CO)_3_Cl}_2_(adc)] resulted in the formation of mononuclear radical complexes of the assumed composition [Re(CO)_3_(solv)(adc)]^•^ which could be seen from six-line patterns (^185,187^Re, *I* = 5/2, Figures S4 and S17) with the exception of the adcpip derivative where we observed 11 lines indicating two Re centres (Figure S2). A similar reactivity was observed for related [Re(CO)_3_Cl(N^N)] complexes containing diimines [47,48,49,50,53,59,66,67,68,69]. We recorded EPR spectra of these radical Re(adc) species in the X-band at 298 K in fluid solution and at high frequencies at 4 K in glassy frozen matrices (see spectra and data in the Supplementary Materials). However, since the underlying species were not clear we refrain from discussing the results here.

**Figure 3:**
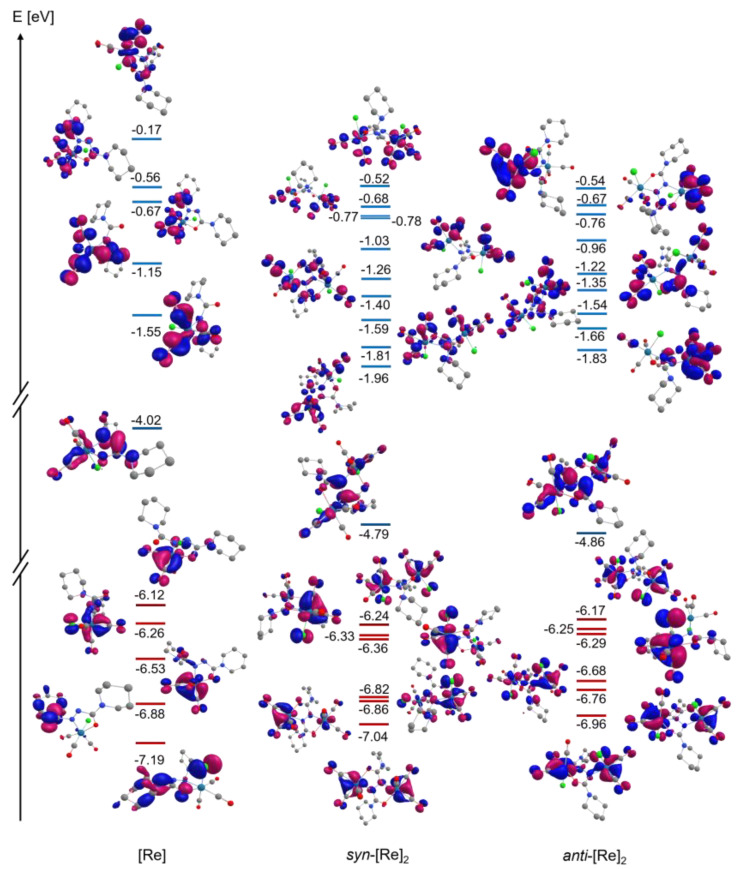
DFT-calculated energies of occupied MOs (red) and unoccupied MOs (blue) with isosurfaces (isovalue 0.04) for complexes [Re(CO)_3_Cl(adcpip)] ([Re]), *syn*-[{Re(CO)_3_Cl}_2_(µ-adcpip)] (*syn*-[Re]_2_) and *anti*-[{Re(CO)_3_Cl}_2_(µ-adcpip)] (*anti*-[Re]_2_); TPSSh/def2-TZVP(+def2-ECP for Re)/CPCMC(THF) level of theory.

**DFT-calculated frontier orbitals:** The DFT-calculated highest occupied molecular orbitals (HOMOs) of the adcpip complexes are localised on the Re centers and exhibit strong d character (Figure 3).

Energetically below for [Re(CO)_3_Cl(adcpip)] ([Re]), occupied orbitals centered on the non-coordinating regions of the adcpip ligand as well as on the chlorido ligand are found. Generally for the dinuclear complexes [{Re(CO)_3_Cl}_2_(µ-adcpip)] (*anti*-[Re]_2_ and *syn*-[Re]_2_), pairs of orbitals which are similar in character but localised on opposite Re centres are observed. Relatedly, the density of energy levels is higher in the dinuclear complexes. The lowest unoccupied molecular orbitals (LUMOs) for both mononuclear [Re(CO)_3_Cl(adcpip)] ([Re]) and dinuclear [{Re(CO)_3_Cl}_2_(µ-adcpip)] (*anti*-[Re]_2_ and *syn*-[Re]_2_) are mostly localised on the NC(O)N=NC(O)N frame of the adcpip ligands with the largest contribution from the azo N=N groups (Figure 3). They also feature significant contributions from the CO ligand in *trans* position to the azo group. At significantly higher energies by about 2.5 eV ([Re]) to 3.0 eV (*anti*-[Re]_2_), multiple unoccupied orbitals localised on the CO ligands are found for all complexes. At even higher energies of −0.17 eV for [Re] and −0.52/−0.54 eV for *syn*-[Re]_2_ and *anti*-[Re]_2_, antibonding orbitals with d_x2-y2_ and d_z2_ character begin to appear. Overall, all unoccupied, antibonding levels are predicted at lower energies in the dinuclear complexes compared to the mononuclear derivative. This is in line with the experimentally observed lower stability and more facile first reduction of the dinuclear complexes. The HOMO–LUMO gap of the mononuclear [Re] is predicted at 2.1 eV, whereas that of the [Re]_2_ isomers is calculated to be around 1.3 to 1.5 eV. This is in excellent agreement with the experimentally observed electrochemical HOMO–LUMO gaps of >1.7 V for [Re] and about 1.3 V for the dinuclear complex, when considering that the calculated values can be expected to be slightly larger as geometry relaxation effects upon oxidation/reduction are neglected.

The calculated LUMO energies for the *syn* and *anti* isomers of [Re]_2_ differ by 0.13 V in favour of the *anti* isomer, but the electrochemical measurements (see above) did not show a splitting of the first reduction wave.

### 2.5. UV–Vis–NIR Absorption Spectroscopy

**Figure 4:**
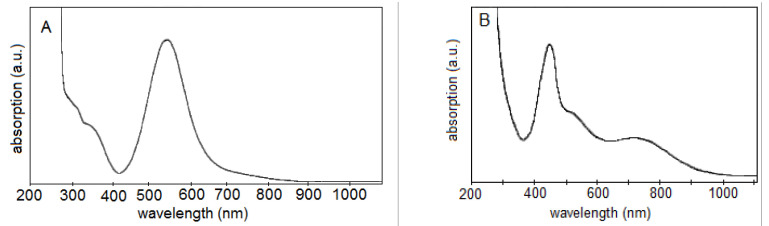
UV–Vis–NIR absorption spectrum of [Re(CO)_3_Cl(µ-adcpip)] (**A**) and [{Re(CO)_3_Cl}_2_(µ-adcpip)] (**B**) in CH_2_Cl_2_.

The UV–Vis–NIR absorption spectra of the three dinuclear [{Re(CO)_3_Cl}_2_(µ-adc)] complexes all show quite intense long-wavelength bands in the range 720 to 850 nm (Figure 4 and Figure S22, data in Table 4), which show pronounced negative solvatochromic behaviour (Table S7). The mononuclear [Re(CO)_3_Cl(µ-adcpip)] shows an intense band centred at 538 nm in keeping with the violet colour, while [Re(CO)_3_Cl(pacOEt)] shows a slightly red-shifted maximum at 502 nm.

This places the adc complexes at the “red end” of the series of dinuclear Re(CO)_3_Cl complexes with bridging diimine ligands (Table 4). The long wavelength bands of the azopyridine (apy), the tetrazine bptz and the pyrazine (bpip) derivatives lie in the same range. Their optical band gaps are all below 2 eV and increase along the series adcOEt < adcO*i*Pr < apy < bptz < adcpip, while the complexes of the established bpip and bpym lie markedly over 2 eV.

**Figure 5:**
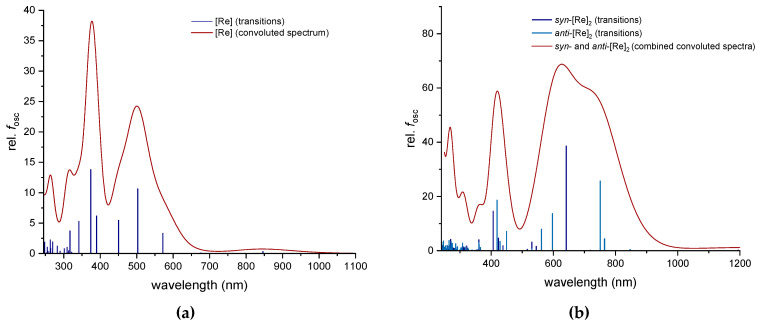
TD-DFT-calculated UV–Vis absorption spectra (**a**) [Re(CO)_3_Cl(adcpip)] ([Re]), (**b**) *syn*-[{Re(CO)_3_Cl}_2_(µ-adcpip)] (*syn*-[Re]_2_) and *anti*-[{Re(CO)_3_Cl}_2_(µ-adcpip)] (*anti-*[Re]_2_); TPSSh/def2-TZVP(+def2-ECP for Re)/CPCMC(THF) level of theory.

The TD-DFT-calculated long-wavelength absorption bands of the dinuclear *anti*-[Re]_2_ and *syn*-[Re]_2_ were found at 585 and 754 nm (*anti*) and 642 nm (*syn*), respectively (Figure 5, data in Tables S8 and S9). Combining the individual spectra of the two *syn* and *anti* isomers gives a broad band with maxima around 700 nm and extending beyond 1000 nm in the NIR range. This prediction is in good agreement with the experimentally observed data, which show a broad band in this region with the maximum at about 730 nm. A second intense band was calculated at around 420 nm, while the experimental spectrum shows this band at about 450 nm. Thus, the calculated spectrum is overall in very good agreement with the experimental data and systematically blue-shifted by only a small offset of ~30 nm.

For the mononuclear [Re], the calculated long-wavelength band lies at 501 nm, while the experimental value was found at 538 nm, which is again a small blue-shift for the calculated data. Further absorption maxima are predicted at 317 and 379 nm, which are visible as shoulders in the experimental data. Overall, the calculated spectrum is also in very good qualitative agreement for [Re]. In addition, both calculated spectra show resolved bands in the UV range, while in the experimental spectra bands in the UV are not resolved and were merged into the solvent UV cutoff.

Trials to monitor the UV–Vis absorption spectra while changing the electrochemical potentials (spectroelectrochemistry) allowed us to confirm for [Re]_2_ that we have indeed isolated the neutral complex and the spectrum in Figure 4 represents [Re]_2_ and not the reduced form. Unfortunately, further efforts to generate the radical anionic complexes (mono- or dinuclear) failed and we observed rapid decomposition for all examples.

In order to obtain an idea why the reduced complexes are so labile, we embarked on calculating the optimised geometries of [Re]^•−^, *anti*-[Re]_2_^•−^ and *syn*-[Re]_2_^•−^.

**Figure 6:**
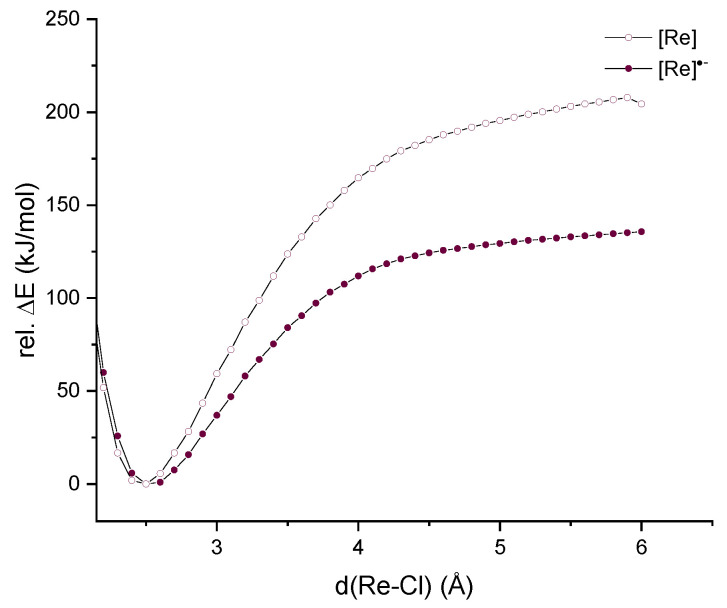
Potential energy surface scan along the Re–Cl vector for [Re] and [Re]^•−^; TPSSh/def2-TZVP(+def2-ECP for Re)/CPCMC(THF) level of theory.

DFT potential energy surface scans along the Re-Cl vector (Figure 6) reveal a markedly labilised Re–Cl bond in [Re(CO)_3_Cl(adcpip)]^n^ upon one-electron reduction from n = 0 to n = −1.

Complex fragmentation along the Re-Cl bond is thus much more favourable in the one-electron reduced anionic state. This redox-dependent weakening of the Re-Cl bond in [Re]^•−^ and the dinuclear [Re]_2_^•−^ complexes is in line with the observed rapid ligand exchange (Cl vs. N donors) observed for these radicals and the irreversible character of the first reduction in the CV. This is also in agreement with the frequent experimental observations of halide loss after reduction in [Re(CO)_3_X] complexes [44,47,48,49,50,59,61,64,65,66,67,68,69].

We further assume that the cleavage of the Re-Cl bond and replacement by solvent molecules obviously trigger the following loss of one [Re(CO)_3_(solv)]^+^ fragment from the reduced dinuclear complexes [Re]_2_^•−^.

## 3. Conclusions

Reactions of [Re(CO)_5_Cl] with the azodicarboxylic esters adcOR (R = Et, *i*Pr, *t*Bu, Bn (CH_2_-C_6_H_5_) and Ph) and the piperidinyl amide derivative adcpip were attempted and led to successful isolation of dinuclear Re(CO)_3_ complexes [{Re(CO)_3_Cl}_2_(µ-adcOR)] (R = Et or *i*Pr) and [{Re(CO)_3_Cl}_2_(µ-adcpip)] in low yields. From the adcpip ligand, the mononuclear, thermally more stable derivative [Re(CO)_3_Cl(adcpip)] was obtained in very good yields. Reacting the dinuclear [{Re(CO)_3_Cl}_2_(µ-adcpip)] with PPh_3_ led to the isolation of the mononuclear [Re(CO)_3_(PPh_3_)(adcpip)]Cl, indicating that the assumedly initially formed dinuclear species [{Re(CO)_3_(PPh_3_)_2_]}_2_(µ-adcpip)]^2+^ is very reactive.

The solid state molecular structure of [Re(CO)_3_Cl(adcpip)] from SC-XRD as well as experimental IR spectra of the mono- and dinuclear adcpip complexes allowed for benchmarking of DFT calculations. Excellent alignment of experimental and calculated results was observed when using def2-TZVP basis sets for all atoms as well as def2-ECP for Re, the BP86 functional for geometry optimisations and the TPSSh hybrid functional for single-point and TD-DFT calculations. Geometry optimisations of the *syn* and *anti* derivatives of the dinuclear [{Re(CO)_3_Cl}_2_(µ-adcpip)] complex predict symmetries and non-planar Re-N=N-Re moieties consistent with steric strain imposed by the two Re(CO)_3_Cl fragments opposing each other on the very small ligand scaffold and with literature reports of similar [Re(CO_3_)Cl(N^N)] fragments.

The expected excellent π-accepting properties are mirrored in the easy reduction of the complexes with redox potentials slightly lower than 0 V vs. ferrocene/ferrocenium, which means that they are positive on the SCE and NHE scales. This is reflected in the observation that solutions of [{Re(CO)_3_Cl}_2_(µ-adcpip)] contain already measurable amounts of a dinuclear radical anionic complex as observed via EPR spectroscopy. The observed potentials agree quite well with the calculated HOMO–LUMO gaps for the adcpip complexes. As expected, the LUMO is centred on the adcpip ligand with large coefficients at the azo group, showing π* character. The low-lying π* orbitals are also responsible for the very long-wavelength transitions observed in the NIR range (700 to 1100 nm).

Trials to record EPR spectra of the reduced complexes failed due to their inherent instability. Only for the dinuclear adcpip complex did we observe an 11-line signal upon electrochemical or chemical reduction in agreement with ligand-based radical with two Re centres of nuclear spin *I* = 5/2 (^185,187^Re). All other attempts led to six-line spectra in agreement with the loss of one Re(CO)_3_ fragment from the dinuclear radical complexes. Assuming Cl- cleavage as the first (rapid) decomposition reaction after reduction, DFT calculations showed that Cl^−^ cleavage is faster for the reduced complexes. DFT potential energy surface scans for the Re-Cl bond in [Re(CO)_3_Cl(adcpip)] and [Re(CO)_3_Cl(adcpip)]^•−^ show a marked labilisation of the Re-Cl bond in line with this assumption.

In the context of possible applications in low-energy electron transfer materials in catalysis or low-energy absorbing materials in optoelectronics, the herein studied adcpip complexes [{Re(CO)_3_Cl}_2_(µ-adc)] are promising with regard to their properties, but not their stability. In future studies, we will thus re-consider the [{Re(CO)_3_Cl}_2_(apy)] complex and investigate its substitution stability and general stability under excitation and electrochemical reduction.

## 4. Experimental Section

**General:** The dialkyl azodicarboxylate ligands adcOEt, adcO*i*Pr, adcO*t*Bu, adcOBn and 1,1′-azodicarbonyl-dipiperidine (adcpip) were supplied from Sigma-Aldrich (Merck, Darmstadt, Germany) and were used without further purification. All syntheses were performed under an inert atmosphere of Ar using standard Schlenk techniques.

### Syntheses

**Starting materials:** [Re(CO)_5_Cl] [70,71,72] and phenylazocarboxylic ethylester (pacOEt)[54] were synthesised according to established procedures. The complex [{Re(CO)_3_Cl}_2_(µ-apy)] was prepared as previously described [53].

Syntheses of the dinuclear complexes [{Re(CO_3_)Cl}_2_(µ-adc)]—general description.

First, 0.5 mmol of the adc ligands was heated with 1.1 mmol (398 mg) [Re(CO)_5_Cl] in a mixture of toluene and CH_2_Cl_2_ (3:1 *v*:*v*) under an Ar atmosphere to 70 °C. After about 30 min, the product formation leads to dark blue-green solutions and after 6 h the reaction was stopped. Further reaction times lead to formation of increasing amounts of the side product [Re_2_(CO)_8_Cl_2_]. After evaporation of the solvents, the dark black materials were re-crystallised from CH_2_Cl_2_/hexane (1:1) and the residue was extracted using toluene and the extracts dried in vacuo.

**[{Re(CO)_3_Cl}_2_(adcOEt)]:** Yield: 78 mg (0.1 mmol, 20%), green-black. C_12_H_10_Cl_2_N_2_O_10_Re_2_ (785.54): calcd. C 18.35, H 1.28, N 3.57; found C 18.32, H 1.24, N 3.55%. NMR spectra could not be recorded due to paramagnetic species (see EPR spectroscopy). IR (CH_2_Cl_2_): 2020, 1917 cm^−1^. EI-MS(+): m/z = 787 [M+H]^+^, 751 [M-Cl]+, 481 [M-(Re(CO)_3_Cl)+H]^+^, 445 [M-(Re(CO)_3_Cl)-Cl]^+^, 175 [adcOEt+H]^+^.

**[{Re(CO)_3_Cl}_2_(adcO*i*Pr)]:** Yield: 106 mg (0.13 mmol, 26%), green-black. C_14_H_14_Cl_2_N_2_O_10_Re_2_ (813.59): calcd. C 20.67, H 1.73, N 3.44; found C 20.66, H 1.74, N 3.46%. NMR spectra could not be recorded due to paramagnetic species (see EPR spectroscopy). IR (toluene): 2022, 1920 cm^−1^. EI-MS(+): m/z = 815 [M+H]^+^, 766 [M-Cl]^+^, 473 [M-(Re(CO)_3_Cl)-Cl]^+^ 203 [adcO*i*Pr+H]^+^.

**[{Re(CO)_3_Cl}_2_(adcpip)]:** For this reaction, a violet intermediate colour was observed in the reaction solution. Yield: 235 mg (0.27 mmol, 54%), green-black. C_18_H_20_Cl_2_N_4_O_8_Re_2_ (863.69): calcd. C 25.03, H 2.33, N 6.49; found C 25.01, H 2.34, N 6.51%. NMR spectra could not be recorded due to paramagnetic species (see EPR spectroscopy). IR (DCE): 2020, 1917 cm^−1^. EI-MS(+): m/z = 864 [M]^+^, 829 [M-Cl]^+^, 523 [M-(Re(CO)_3_Cl)-Cl]^+^, 253 [adcpip+H]^+^.

**Synthesis of [Re(CO)_3_Cl(adcpip)]:** 180 mg [Re(CO)_5_Cl] (0.5 mmol) and 126 mg adcpip (0.5 mmol) were dissolved in 30 mL toluene and 10 mL CH_2_Cl_2_ and heated to 90 °C. The yellow solution turned violet within 10 min and the reaction was stopped after 6 h upon which a dark violet precipitate had formed. The material was filtered off and recrystallised from CH_2_Cl_2_/n-heptane (2:1) to yield deep purple microcrystalline materials which were dried in vacuo. Yield: 245 mg (0.44 mmol, 88%), dark violet. C_15_H_20_ClN_4_O_8_Re (558.03): calcd. C 32.29, H 3.61, N 10.04; found C 32.30, H 3.63, N 10.01%.1H NMR (300 MHz, CDCl_3_): *δ* = 4.34–3-13 (m, 8H, H1,H5), 2.00–1.50 (m, 12H, H2,H3,H4). ^13^C NMR (75 MHz, CDCl_3_): δ = 47.0, 46.9 (pip1,5), 25.8, 25.5, 24.0, 23.8 (pip2,3,4), the carbonyl C were not detected. IR (DCE): 2040, 1960, 1920 cm^−1^. EI-MS(+): m/z = 559 [M+H]^+^, 523 [M-Cl]^+^, 253 [adcpip+H]^+^.

**Attempted synthesis of [(Re(CO)_3_(PPh_3_)}_2_(µ-adcpip)]Cl_2_:** 87 mg (0.1 mmol) of [{Re(CO)_3_Cl}_2_(adcpip)] was dissolved 20 mL 1,2-dichloroethane and 52 mg (0.2 mmol) of PPh_3_ was added and the reaction mixture turned brown. EPR spectroscopy of this solution gave strong indication of a mononuclear radical complex. The mixture was stirred for another 30 min, and then all volatiles were evaporated. Dissolving the material in CH_2_Cl_2_ and careful precipitation using about 5× the volume of *n*-hexane gave 26 mg of brown material. Elemental analyses for the assumed mononuclear [Re(CO)_3_(PPh_3_)(adcpip)]Cl C_33_H_35_N_4_O_5_PRe (784.84): calcd. C 50.50, H 4.50, N 7.14; found C 50.45, H 4.54, N 7.11%. IR (CH_2_Cl_2_): 2010s, 1910vs, 1876vs 1778vs, 1720vs. EI-MS(+): m/z = 785 [M]^+^, 253 [adcpip+H]^+^.

**Synthesis of [Re(CO)_3_Cl(pacOEt)]:** 70 mg (0.39 mmol) phenylazocarboxylic ethylester (pacOEt) was heated with 142 mg (0.39 mmol) [Re(CO)_5_Cl] in 25 mL of toluene and CH_2_Cl_2_ (3:1 *v*:*v*) under an inert atmosphere of argon to 80 to 90 °C for 13 h, yielding a red solution. After evaporation of the solvents, the resulting dark red material was extracted using toluene and the extracts dried in vacuo. Yield: 142 mg (0.29 mmol, 75%), red-violet. C_12_H_10_ClN_2_O_5_Re (483.88): calcd. C 29.79, H 2.08, N 5.79; found C 29.75, H 2.06, N 5.74%. IR (DCE): 2022, 1962, 1928 cm^−1^. EI-MS(+): m/z = 485 [M+H]^+^, 449 [M-Cl]^+^, 179 [pacOEt+H]^+^.

**Characterisation of [(CO)_4_Re(µ-Cl_2_)Re(CO)_4_]:** The colourless material was obtained from several reaction mixtures of [Re(CO)_5_Cl] and the adc ligands and was isolated from the grey residues after extraction with toluene. Careful recrystallisation from CH_2_Cl_2_ yielded colourless crystals. Yields ranged from 42 to 100% of the original Re contents. C_8_Cl_2_O_8_Re_2_ (667.39): calcd. C 14.40, Cl 10.62; found C 14.40, Cl 10.61%. IR (solid): 2118, 2019, 1995, 1928 cm^−1^.

**Instrumentation:** NMR spectra were recorded with a Bruker Avance II 300 MHz spectrometer (^1^H: 300.13 MHz) equipped with a BBO ATM 5 mm probe head with z-gradient (Bruker, Rheinhausen, Germany). Chemical shifts were relative to TMS. The spectral analyses were performed by the BrukerTopSpin 2 software. Elemental analyses were carried out using a Hekatech CHNS EuroEA 3000 Analyzer (Hekatech, Wegberg, Germany). The Cl contents of [(CO)_4_Re(µ-Cl_2_)Re(CO)_4_] were obtained through dissolution using HNO_3_ and aqueous determination of Cl^−^. IR spectra were measured in ATR mode using a Perkin Elmer 400 or a Thermo Avatar 370 DTGS FT-IR spectrometer (Perkin Elmer, Rodgau, Germany). UV–Vis absorption spectra were recorded on a Varian Cary50 Scan spectrophotometer (Varian, Darmstadt, Germany). EPR spectra were recorded in the X-band with a Bruker System ESP300 or ELEXSYS 500E, equipped with a Bruker Variable Temperature Unit ER 4131VT (Bruker, Rheinhausen, Germany). *g* values were calibrated using a dpph sample. Spectral simulation was performed using Bruker SimFonia V1.26. Electrochemical experiments were carried out in 0.1 M *n-*Bu_4_NPF_6_ solutions using a three-electrode configuration (glassy carbon working electrode, Pt counter electrode, Ag/AgCl pseudo reference) and an EG&G Parc model 175 (EG&G, Gaithersburg, MD, USA), a Metrohm Autolab PGSTAT30 or a Metrohm µStat400 potentiostat (Metrohm, Filderstadt, Germany). Experiments were run at a scan rate of 100 mV/s at ambient temperature, the ferrocene/ferrocenium couple served as internal reference. UV–Vis–spectroelectrochemical measurements in 0.1 M *n*Bu_4_NPF_6_/CH_2_Cl_2_ solution were performed using an optically transparent thin-layer electrode (OTTLE) cell [73,74] at room temperature.

**Computational details:** Calculations were performed using the software package ORCA 5.0.2 [75]. Geometry optimisations and subsequent numerical frequency calculations were performed using the BP86 functional [76,77], Grimme’s D3 dispersion correction [78,79], def2-TZVP basis sets for all elements with def2-ECP for Re [80] and CPCMC(THF) as an approximate solvent model [81]. On the optimised geometries, single-point and TD-DFT/TDA [82,83] calculations were performed using the TPSSh functional [84], Grimme’s D3 dispersion correction, def2-TZVP basis sets for all elements with def2-ECP for Re and CPCMC(THF) like before. The output concerning geometries, IR frequencies, molecular orbitals and electronic transitions was analysed and visualised using the built-in ORCA modules orca_plot and orca_mapspc in combination with the software packages Chemcraft [85] and Origin 2021 [86].

The same properties were also calculated using the M06-2X functional [87], LANL2DZ with the corresponding Los Alamos ECP for Re [88,89,90] and def2-TZVP basis sets for all other atoms [80] and CPCM(THF) within the Gaussian program package [91].

**Single crystal X-ray diffraction:** SC-XRD measurements were performed at 170(2) K, employing a Bruker D8 Venture diffractometer including a Bruker Photon 100 CMOS detector using Mo K_α_ radiation (λ = 0.71073 Å). The crystal data were collected using APEX4 v2021.10-0 [92]. The structure was solved by dual space methods using SHELXT, and the refinement was carried out with SHELXL employing the full-matrix least-squares methods on F_O_^2^ < 2σ(F_O_^2^) as implemented in ShelXle [93,94,95]. The non-hydrogen atoms were refined with anisotropic displacement parameters without any constraints. The hydrogen atoms were included by using appropriate riding models. Data of the structure solutions and refinement of [Re(CO)_3_Cl(adcpip)] can be obtained free of charge at https://www.ccdc.cam.ac.uk/structures/ (accessed on 3 November 2022) under the deposition number 2194078, or from the Cambridge Crystallographic Data Centre, 12 Union Road, Cambridge, CB2 1EZ UK (Fax: +44-1223-336-033 or e-mail: deposit@ccdc.cam.ac.uk).

## Figures and Tables

**Table 1:** Characteristic experimental IR frequencies of Re complexes and calculated data ^a^.

Compound	Exp.				Calc. (THF)		
	νCO	(cm^−1^)		Solvent			
Band	I	II	III		I	II	III
[Re(CO)_3_Cl(adcpip)]	2040	1960	1920	DCE	2007 ^b^	1927 ^b^	1894 ^b^
[Re(CO)_3_(PPh_3_)(adcpip)]Cl	2010	1910	1876	DCE			
[Re(CO)_3_Cl(pacOEt)]	2022	1962	1928	DCE			
*syn*-[{Re(CO)_3_Cl}_2_(µ-adcpip)]	2015	1913	^c,d^	DCE	1978 ^b^	1932, 1929 ^b^	1903, 1895 ^b^
*anti*-[{Re(CO)_3_Cl}_2_(µ-adcpip)]					1981 ^b^	1938, 1927 ^b^	1908, 1895 ^b^
[{Re(CO)_3_Cl}_2_(µ-adcOEt)]	2019	1916		DCE			
[{Re(CO)_3_Cl}_2_(µ-adcO*i*Pr)]	2021	1919		DCE			
[Re(CO)_3_Cl(bpy)] ^e^	2024	1917	1900	MeCN			
[Re(CO)_3_Cl(bpip)] ^f^	2027	1934	1905	THF			
[{Re(CO)_3_Cl}_2_(µ-apy)] ^g^	2005	1952	1938	THF			
[{Re(CO)_3_Cl}_2_(µ-bptz)] ^h^	2010	1950	1921	Acetone			
[{Re(CO)_3_Cl}_2_(µ-bpip)] ^f^	2020	1940	1910	THF			
[{Re(CO)_3_Cl}_2_(µ-bpym)] ^i^	2025	1938	1900	Acetone			

^a^ DCE = 1,2-dichloroethane. From calculations using BP86/def2-TZVP(+def2-ECP for Re)/CPCMC(THF). ^b^ All bands shifted by about 35 cm^−1^ compared with the experimental data. ^c^ A closer inspection showed several maxima and shoulder at: 2036sh, 2015, 1970sh, 1930sh, 1913, 1872sh in line with the five calculated maxima. ^d^ Three resonances were observed in toluene/CH_2_Cl_2_: 2015, 1939, 1912 cm^−1^. ^e^ bpy = 2,2′-bipyridine, from Ref. [59]. ^f^ bpip = 2,5-bis(1-phenyliminoethyl)pyrazine, from Ref. [50]. ^g^ apy = 2,2′-azobipyridine, from Refs. [49,52,53]. ^h^ bptz = 2,5-bis(2-pyridyl)-1,3,4,6-tetrazine, from Ref. [53]. ^i^ bpym = 2,2′-bipyrimidine, from Refs. [52,53].

**Table 2:** Selected metrics from the crystal structure of [Re(CO)_3_Cl(adcpip)] (exp.) compared to DFT-calculated data (calc.) ^a^.

Bond Lengths (Å)			Angles (°)		
	Exp.	Calc.		Exp.	Calc.
Re1–Cl1	2.457(1)	2.473	Cl1–Re1–C1	174.5(1)	177.4
Re1–C1	1.924(2)	1.933	Cl1–Re1–C2	95.6(1)	93.6
Re1–C2	1.897(2)	1.912	Cl1–Re1–C3	87.0(1)	88.6
Re1–C3	1.942(2)	1.969	Cl1–Re1–O4	81.6(1)	81.9
Re1–O4	2.151(1)	2.169	Cl1–Re1–N1	89.6(1)	90.0
Re1–N1	2.117(2)	2.078	O4-Re1-N1	71.6(1)	72.1
N1–N2	1.261(2)	1.283	O4–Re1–C1	95.2(1)	96.9
C1C–O4	1.257(3)	1.275	O4–Re1–C2	170.2(1)	169.4
C2C–O5	1.211(3)	1.227	O4–Re1–C3	100.9(1)	98.5
N1–C2C	1.489(2)	1.484	C1–Re1–C2	88.3(1)	87.9
N2–C1C	1.443(2)	1.414	C1–Re1–C3	89.2(1)	89.3
C1–O1	1.148(3)	1.164	C1–Re1–N1	93.7(1)	91.8
C2–O2	1.156(3)	1.167	C2–Re1–C3	88.3(1)	91.0
C3–O3	1.145(3)	1.157	C2–Re1–N1	99.0(1)	98.3
			C3–Re1–N1	172.2(1)	170.7
			N2–N1–Re1	123.1(1)	122.7
Dihedral angles (°)					
Re1–N1–N2–C1C	0.9(2)	2.9	N1–N2–C1C–N3	176.5(2)	173.5
C2C–N1–N2–C1C	172.9(2)	168.2	N2–N1–C2C–N4	99.8(2)	102.8

^a^ At BP86/def2-TZVP(+def2-ECP for Re)/CPCMC(THF) level of theory.

**Table 3:** Electrochemical data of adc ligands and Re complexes ^a^.

Compound	*E_1_*_/*2*_ Red1	*E_pc_* Red2	Epa Ox1	Ref.
[Re(CO)_3_Cl(adcpip)]	−0.50	−1.18	>1.2	this work
[{Re(CO)_3_Cl}_2_(µ-adcpip)]	−0.20	−1.48	1.12	this work
*syn*-[{Re(CO)_3_Cl}_2_(µ-adcOEt)] ^b^	−0.05	−0.94	1.17	this work
*anti*-[{Re(CO)_3_Cl}_2_(µ-adcOEt)] ^b^	−0.16	−0.95	1.17	this work
[{Re(CO)_3_Cl}_2_(µ-adcO*i*Pr)]	−0.17	−0.99	1.13	this work
[Re(CO)_3_Cl(pacOEt)]	−0.77	−1.58	1.11	this work
[Re_2_(µ-Cl)_2_(CO)_8_]	−2.31	−2.68	1.32	this work
[Re(CO)_3_Cl(bpy)] ^b^	−1.74	−2.20		[59]
[Re(CO)_3_Cl(bpip)] ^c^	−1.17	−1.89		[50]
[Re(CO)_3_Cl(apy)] ^d^	−0.78	−1.54		[47]
*anti*-[{Re(CO)_3_Cl}_2_(µ-apy)] ^d^	0.00	−0.81		[44]
*syn*-[{Re(CO)_3_Cl}_2_(µ-apy)] ^d^	0.53	−0.63		[44]
[{Re(CO)_3_Cl}_2_(µ-bptz)]	−0.16	−1.49		[53]
[{Re(CO)_3_Cl}_2_(µ-bpip)]	−0.54	−1.30	0.76	[50]
[{Re(CO)_3_Cl}_2_(µ-bpym)] ^d^	0.02.	−0.71	0.73	[53]

^a^ Potentials in V vs. ferrocene/ferrocenium, recorded in 0.1 M *n-*Bu_4_NPF_6_/DCE, half-wave potentials *E_1_*_/*2*_ for reversible waves, *E_pc_* = cathodic peak potential and *E*_pa_ anodic peak potential for irreversible waves, scan rate = 100 mV/s. Conversion of SCE values to ferrocene/ferrocenium = −0.49 V in *n-*Bu_4_NPF_6_/DCE. ^b^ Recorded in DMF. ^c^ In THF. ^d^ In CH_2_Cl_2_.

**Table 4:** Selected long-wavelength UV–Vis absorption maxima of Re complexes ^a^.

Compound	*λ*_1_	*λ*_2_	*λ*_3_	*E*_opt_ Max	Solvent	Ref.
[Re(CO)_3_Cl(adcpip)]			538	2.30	CH_2_Cl_2_	
[Re(CO)_3_Cl(pacOEt)]			502	2.47	CH_2_Cl_2_	
[{Re(CO)_3_Cl}_2_(µ-adcpip)]	452	510	718	1.73	CH_2_Cl_2_	
[{Re(CO)_3_Cl}_2_(µ-adcOEt)]			853	1.45	CH_2_Cl_2_	
[{Re(CO)_3_Cl}_2_(µ-adcO*i*Pr)]		690	837	1.48	CH_2_Cl_2_	
[{Re(CO)_3_Cl}_2_(µ-apy)]		705	784	1.58	CH_2_Cl_2_	[44]
[{Re(CO)_3_Cl}_2_(µ-apy)]		588	747	1.66	DMF	[44]
[{Re(CO)_3_Cl}_2_(µ-bptz)]		510	697	1.78	acetone	[53]
[{Re(CO)_3_Cl}_2_(µ-bptz)]		550	732	1.69	DCE	[53]
[{Re(CO)_3_Cl}_2_(µ-bpip)]		366	584	2.12	acetone	
[{Re(CO)_3_Cl}_2_(µ-bpip)]		378	636	1.95	DCE	[50]
[{Re(CO)_3_Cl}_2_(µ-bpym)		357	469	2.64	acetone	[53]

^a^ Absorption maxima λ in nm, *E*_opt_ = optical band gap in eV.

## Data Availability

Available from the authors on request.

## Supplementary Materials

The following supporting information can be downloaded at: https://www.mdpi.com/article/10.3390/molecules27238159/s1, Figure S1: Part of the IR spectrum recorded on the reaction mixture of [Re(CO)_5_Cl] and adcpip; Figure S2: X-band EPR spectrum of the assumed [{Re(CO)_3_Cl}_2_(µ-adcpip)]^•−^ in toluene/CH_2_Cl_2_; Figure S3: IR spectrum and X-band (9.862 GHz) EPR spectrum of the reaction mixture of [{Re(CO)_3_Cl}_2_(µ-adcpip)] with 2 equivalents of PPh_3_ in THF; Figure S4: X-band EPR spectrum of the assumed [{Re(CO)_3_Cl}_2_(µ-adcpip)]^•−^ in toluene before and after addition of a small amount of MeCN; Figure S5: 300 MHz ^1^H NMR spectra of adcpip and [Re(CO)_3_Cl(adcpip)]; Figure S6: 75 MHz ^13^C DEPTQ NMR spectrum of [Re(CO)_3_Cl(adcpip)]; Figure S7: IR spectra of [Re(CO)_3_Cl(adcpip)], [Re(CO)_3_(PPh_3_)(adcpip)]Cl, and [Re(CO)_4_(µ-Cl)_2_Re(CO)_4_]; Figure S8: DFT-calculated IR spectra of [Re(CO)_3_Cl(adcpip)] [Re], and [{Re(CO)_3_Cl}_2_(µ-adcpip)] (*anti*-[Re]_2_, and *syn*-[Re]_2_); at TPSSh(def2-TZVP(+def2-ECP for Re)/(CPCMC(THF))) level of theory; Figure S9: DFT-calculated IR spectra of [Re(CO)_3_Cl(adcpip)] [Re], and [{Re(CO)_3_Cl}_2_(µ-adcpip)] (*anti*-[Re]_2_, and *syn*-[Re]_2_); at M06-2X/def2TZVP/LANL2DZ/CPCM(THF) level of theory; Figure S10: Views on the crystal structure of [Re(CO)_3_Cl(adcpip)]; Figure S11: Views on the DFT-optimised structures in the *S_0_* ground state for [{Re(CO)_3_Cl}_2_(µ-adcpip)]; M06-2X/def2TZVP/LANL2DZ/CPCM(THF) level of theory; Figure S12: Cyclic voltammograms of [{Re(CO)_3_Cl}_2_(adc-OEt)]; Figure S13: Cyclic voltammogram of [{Re(CO)_3_Cl}_2_(µ-adcpip)]; Figure S14: Cyclic voltammogram of [Re(CO)_3_(PPh_3_)(adcpip)]Cl; Figure S15: Cyclic voltammograms of [Re(CO)_3_Cl(pacOEt)]; Figure S16: Cyclic voltammograms of [Re_2_(µ-Cl)_2_(CO)_8_]; Figure S17: X-band EPR spectra of the assumed [Re(CO)_3_(CH_2_Cl_2_)(adcpip)]^•^ and [Re(CO_3_)(NEt_3_)(adcpip)]^•^ at 298 K; Figure S18: X-band EPR spectra of the assumed [Re(CO)_3_Cl(adcpip)]^•−^ in glassy frozen acetone matrix at 4 K. Figure S19: X-band EPR spectra of the assumed [Re(CO)_3_Cl(adcOEt)]^•−^ (A) and [Re(CO)_3_Cl(adcO*i*Pr)]^•−^ (B) in glassy frozen acetone matrix 4 K. Figure S20: DFT-calculated energies of occupied MOs and unoccupied MOs for the Re complexes [Re], *anti*-[Re]_2_, and *syn*-[Re]_2_; M06-2X/def2TZVP/LANL2DZ for Re/CPCM(THF) level of theory; Figure S21: DFT-calculated frontier orbital landscape in the ground state (*S_0_*) for [Re], *anti*-[Re]_2_, and *syn*-[Re]_2_; M062X/def2TZVP/LANL2DZ/CPCM(THF) level of theory; Figure S22: UV–Vis–NIR absorption spectrum of [{Re(CO)_3_Cl}_2_(µ-adcO*i*Pr)] and [{Re(CO)_3_Cl}_2_(µ-adcOEt)]; Figure S23: TD-DFT-calculated UV–Vis absorption spectra (A): overlay spectra of [Re], *anti*-[Re]_2_ and *syn*-[Re]_2_; (B): [Re]; (C): *anti*-[Re]_2_; (D): *syn*-[Re]_2_; M06-2X/def2TZVP/LANL2DZ for Re/CPCM(THF) level of theory; Figure S24: Views on the DFT-optimised structures in the *D_0_* ground state for [Re]^•−^, *anti*-[Re]_2_^•−^ and *syn*-[Re]_2_^•−^; at BP86/def2-TZVP(+def2-ECP for Re)/CPCMC(THF) level of theory; Figure S25: DFT-calculated frontier orbital landscape in the ground state (*D_0_*) for [Re]^•−^ and *anti*-[Re]_2_^•−^; TPSSh/def2-TZVP(+def2-ECP for Re)/CPCMC(THF) level of theory; Table S1: Crystal structure and solution data of [Re(CO)_3_Cl(adcpip)]; Table S2: Selected metrics from the crystal structure of [Re(CO)3Cl(adcpip)]; Table S3A: Selected DFT-calculated metrics of [Re], *anti*-[Re]_2_ and *syn*-[Re]_2_, compared with [Re]^•−^, *anti*-[Re]_2_^•−^ and *syn*-[Re]_2_^•−^; at BP86/def2-TZVP(+def2-ECP for Re)/CPCMC(THF) level of theory; Table S3B: Selected DFT-calculated metrics of [Re], *anti*-[Re]_2_ and *syn*-[Re]_2_; at M06-2X/ def2TZVP/LANL2DZ/CPCM(THF) level of theory; Table S4: Experimental IR data of adc ligands and Re complexes; Table S5: Electrochemical data of adc ligands; Table S6: Selected X-band EPR data of reduced Re complexes; Table S7: UV–Vis long-wavelength absorption maxima of [{Re(CO)_3_Cl}_2_(µ-adcpip)] in different solvents; Table S8: DFT-calculated electronic transitions and character thereof for [Re]; TPSSh/def2-TZVP(+def2-ECP for Re)/CPCMC(THF) level of theory; Table S9: DFT-calculated electronic transitions and character thereof for *anti*-[Re]_2_; TPSSh/def2-TZVP(+def2-ECP for Re)/CPCMC(THF) level of theory; Table S10: DFT-calculated electronic transitions and character thereof for *syn*-[Re]_2_; TPSSh/def2-TZVP(+def2-ECP for Re)/CPCMC(THF) level of theory; Table S11: DFT-calculated absorptions and character of calculated transitions for [Re]; M06-2X/def2TZVP/LANL2DZ for Re/CPCM(THF) level of theory; Table S12: DFT-calculated absorptions and character of calculated transitions for *anti*-[Re]_2_; M06-2X/def2TZVP/LANL2DZ for Re/CPCM(THF) level of theory. Table S13: DFT-calculated absorptions and character of calculated transitions for *syn*-[Re]_2_; M06-2X/def2TZVP/LANL2DZ for Re/CPCM(THF) level of theory.

## Sample Availability

Samples of the compounds are available from the authors.
