# Supplementary material for: Rhenium Tricarbonyl Complexes of Azodicarboxylate Ligands
Source: Molecules. 2022 Nov 23;27(23):8159. doi: 10.3390/molecules27238159 (PMC9740152; doi:10.3390/molecules27238159)
Supplement: Supplementary file 1 [file molecules-27-08159-s001.zip › molecules-2019093-supplementary.pdf]

Rhenium Tricarbonyl Complexes of Azodicarboxylate Ligands

Rose Jordan [1], Maryam Niazi [1], Sascha Schäfer [1] and Wolfgang Kaim [2] and Axel Klein [1,*]

1 Department für Chemie, Institut für Anorganische Chemie, Mathematisch-Naturwissenschaftliche Fakultät, Universität zu Köln, Greinstrasse 6, D-50939 Köln, Germany
2 Institut für Anorganische Chemie, Universität Stuttgart, Pfaffenwaldring 55, D-70550 Stuttgart, Germany
* Correspondence: axel.klein@uni-koeln.de; Tel.: +49-221-470-4006

Contents

Figure S1. Part of the IR spectrum recorded on the reaction mixture of [Re(CO)$_5$Cl] and adcpip.
Figure S2. X-band EPR spectrum of the assumed [{Re(CO)$_3$Cl}$_2$(μ-adcpip)]$^{\bullet-}$ in toluene/CH$_2$Cl$_2$ observed in thereaction mixture of [Re(CO)$_5$Cl] and adcpip with simulation.
Figure S3. IR spectrum and X-band (9.862 GHz) EPR spectrum of the reaction mixture of [{Re(CO)$_3$Cl}$_2$(μ-adcpip)] with 2 equivalents of PPh$_3$ in THF.
Figure S4. X-band EPR spectrum of the assumed [{Re(CO)$_3$Cl}$_2$(μ-adcpip)]$^{\bullet-}$ in toluene before (A) and after (B) addition of a small amount of MeCN.
Figure S5. 300 MHz ^{1}H NMR spectra of adcpip (top) and [Re(CO)$_3$Cl(adcpip)] (bottom) in CDCl$_3$.
Figure S6. 75 MHz ^{13}C DEPTQ NMR spectrum of [Re(CO)$_3$Cl(adcpip)] in CDCl$_3$.
Figure S7. IR spectra of [Re(CO)$_3$Cl(adcpip)], [Re(CO)$_3$(PPh$_3$)(adcpip)]Cl, and [Re(CO)$_4$(μ-Cl)$_2$Re(CO)$_4$].
Figure S8. DFT-calculated IR spectra of [Re(CO)$_3$Cl(adcpip)] [Re], and [{Re(CO)$_3$Cl}$_2$(μ-adcpip)] (*anti*-[Re]$_2$, and *syn*-[Re]$_2$); at TPSSh(def2-TZVP(+def2-ECP for Re)/(CPCMC(THF) level of theory.
Figure S9. DFT-calculated IR spectra of [Re(CO)$_3$Cl(adcpip)] [Re], and [{Re(CO)$_3$Cl}$_2$(μ-adcpip)] (*anti*-[Re]$_2$,and *syn*-[Re]$_2$); at M06-2X/def2TZVP/LANL2DZ/CPCM(THF) level of theory.
Figure S10. View on the crystal structure of [Re(CO)$_3$Cl(adcpip)] along the crystallographic *b* axis.
Figure S11. Views on the DFT-optimised structures in the S_0 ground state for [{Re(CO)$_3$Cl}$_2$(μ-adcpip)]; M06-2X/def2TZVP/LANL2DZ/CPCM(THF) level of theory.
Figure S12. Cyclic voltammogramms of [{Re(CO)$_3$Cl}$_2$(adc-OEt)] in 0.1 M *n*-Bu$_4$NPF$_6$/DCE.
Figure S13. Cyclic voltammogramm of [{Re(CO)$_3$Cl}$_2$(μ-adcpip)] in *n*-BuNPF$_6$/DCE.
Figure S14. Cyclic voltammogramm of [Re(CO)$_3$(PPh$_3$)(adcpip)]Cl in *n*-BuNPF$_6$/DCE.
Figure S15. Cyclic voltammogramms of [Re(CO)$_3$Cl(pacOEt)] in 0.1 M *n*-Bu$_4$NPF$_6$/DCE.
Figure S16. Cyclic voltammogramms of [Re$_2$(μ-Cl)$_2$(CO)$_8$] in 0.1 M *n*-Bu$_4$NPF$_6$/DCE.
Figure S17. X-band EPR spectra of the assumed [Re(CO)$_3$(CH$_2$Cl$_2$)(adcpip)]$^\bullet$ and [Re(CO$_3$)(NEt$_3$)(adcpip)]$^\bullet$ at 298K.
Figure S18. X-band EPR spectra of the assumed [Re(CO)$_3$Cl(adcpip)]$^{\bullet-}$ in glassy frozen acetone matrix at 4 K.
Figure S19. X-band EPR spectra of assumed [Re(CO)$_3$Cl(adcOEt)]$^{\bullet-}$ (A) and [Re(CO)$_3$Cl(adcO*i*Pr)]$^{\bullet-}$ (B) in glassy frozen acetone matrix 4 K.
Figure S20. DFT calculated energies of occupied MOs (blue) and unoccupied MOs (red) for the Re complexes [Re], *anti*-[Re]$_2$, and *syn*-[Re]$_2$; M06-2X/def2TZVP/LANL2DZ for Re/CPCM(THF) level of theory.
Figure S21. DFT-calculated frontier orbital landscape in the ground state (S_0) for [Re], *anti*-[Re]$_2$, and *syn*-[Re]$_2$; M062X/def2TZVP/LANL2DZ/CPCM(THF) level of theory.
Figure S22. UV-vis-NIR absorption spectrum of [{Re(CO)$_3$Cl}$_2$(μ-adcO*i*Pr)] (left) and [{Re(CO)$_3$Cl}$_2$(μ-adcOEt)] (right) in CH$_2$Cl$_2$.
Figure S23. TD-DFT-calculated UV-vis absorption spectra **A:** Overlay spectra of [Re], *anti*-[Re]$_2$, and *syn*-[Re]$_2$; **B:** [Re]; **C:** *anti*-[Re]$_2$; **D:** *syn*-[Re]$_2$; M06-2X/def2TZVP/LANL2DZ for Re/CPCM(THF) level of theory.
Figure S24. Views on the DFT-optimised structures in the D_0 ground state for [Re]$^{\bullet-}$, *anti*-[Re]$_2{}^{\bullet-}$, and *syn*-[Re]$_2{}^{\bullet-}$; at BP86/def2-TZVP(+def2-ECP for Re)/CPCMC(THF) level of theory.
Figure S25. DFT-calculated frontier orbital landscape in the ground state (D_0) for [Re]$^{\bullet-}$ and *anti*-[Re]$_2{}^{\bullet-}$; TPSSh/def2-TZVP(+def2-ECP for Re)/CPCMC(THF) level of theory.

Table S1. Crystal Structure and solution data of [Re(CO)$_3$Cl(adcpip)].
Table S2. Selected metrics from the crystal structure of [Re(CO)$_3$Cl(adcpip)].
Table S3A. Selected DFT-calculated metrics of [Re], *anti*-[Re]$_2$ and *syn*-[Re]$_2$, compared with [Re]$^{•–}$, *anti*-[Re]$_2$$^{•–}$ and *syn*-[Re]$_2$$^{•–}$; at BP86/def2-TZVP(+def2-ECP for Re)/CPCMC(THF) level of theory.
Table S3B. Selected DFT-calculated metrics of [Re], *anti*-[Re]$_2$ and *syn*-[Re]$_2$; at M06-2X/ def2TZVP/LANL2DZ/CPCM(THF) level of theory.
Table S4. Experimental IR data of adc ligands and Re complexes.
Table S5. Electrochemical data of adc ligands.
Table S6. Selected X-band EPR data of reduced Re complexes.
Table S7. UV-vis long-wavelength absorption maxima of [{Re(CO)$_3$Cl}$_2$(μ-adcpip)] in different solvents.
Table S8. DFT-calculated electronic transitions and character thereof for [Re]; TPSSh/def2-TZVP(+def2-ECP for Re)/CPCMC(THF) level of theory
Table S9. DFT-calculated electronic transitions and character thereof for *anti*-[Re]$_2$; TPSSh/def2-TZVP(+def2-ECP for Re)/CPCMC(THF) level of theory.
Table S10. DFT-calculated electronic transitions and character thereof for *syn*-[Re]$_2$; TPSSh/def2-TZVP(+def2-ECP for Re)/CPCMC(THF) level of theory.
Table S11. DFT-calculated absorptions and character of calculated transitions for [Re]; M06-2X/def2TZVP/LANL2DZ for Re/CPCM(THF) level of theory.
Table S12. DFT-calculated absorptions and character of calculated transitions for *anti*-[Re]$_2$; M06-2X/def2TZVP/LANL2DZ for Re/CPCM(THF) level of theory.
Table S13. DFT-calculated absorptions and character of calculated transitions for *syn*-[Re]$_2$; M06-2X/def2TZVP/LANL2DZ for Re/CPCM(THF) level of theory.

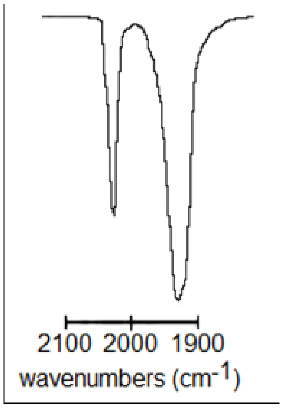

Figure S1. Part of the IR spectrum recorded on the reaction mixture of [Re(CO)$_5$Cl] and adcpip.

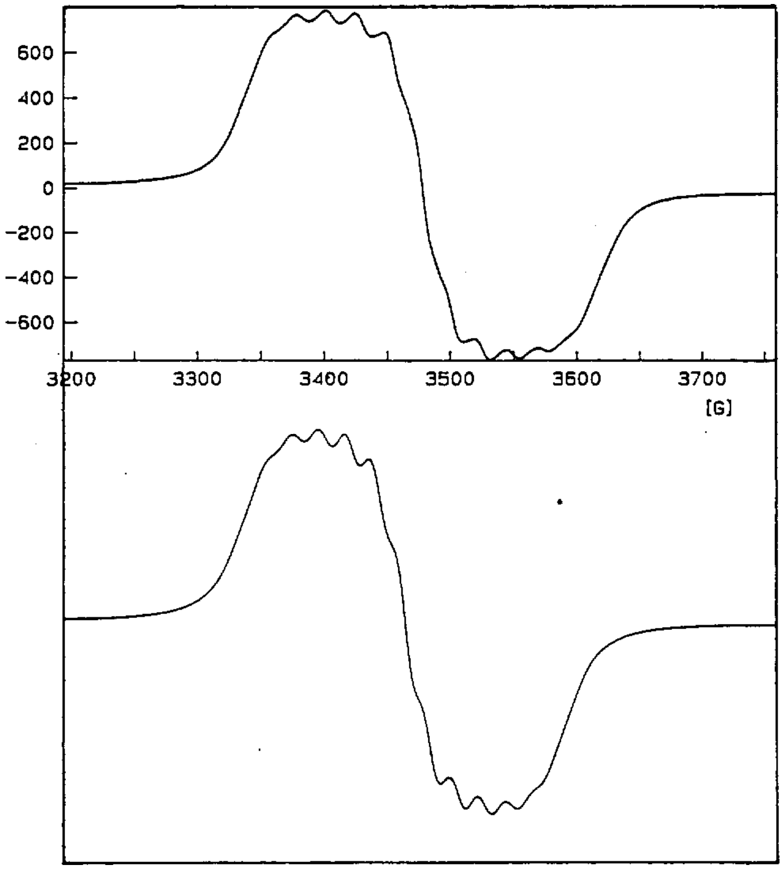

Figure S2. X-band EPR spectrum of the assumed [{Re(CO)$_3$Cl}$_2$(μ-adcpip)]$^{\bullet-}$ in toluene/CH$_2$Cl$_2$ (top) observed in the reaction mixture of [Re(CO)$_5$Cl] and adcpip, measured at 9.863 GHz and 5.0 G modulation amplitude; simulation using A_{Re} = 22.2 G, linewidth = 18.6 G and Lorentzian lines (bottom). Due to rapid Cl$^-$ cleavage, the radical could also be a [{Re(CO)$_3$(solv)}$_2$(μ-adcpip)]$^{\bullet+}$ species.

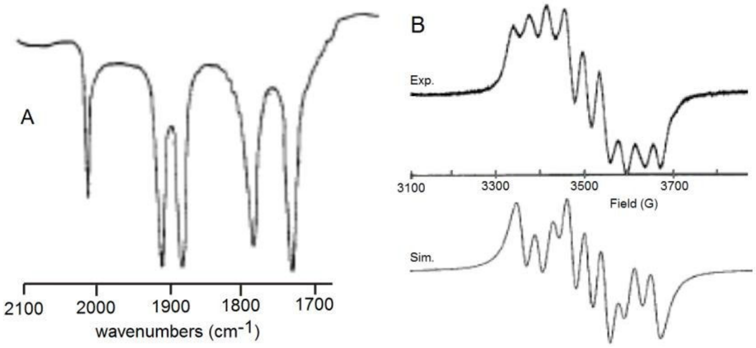

Figure S3. (**A**) IR spectrum and (**B**) X-band (9.862 GHz) EPR spectrum of the reaction mixture of $[\{Re(CO)_3Cl\}_2(\mu\text{-adcpip})]$ with 2 equivalents of PPh_3 in THF. EPR spectrum recorded after 10 min, simulation with one A_{Re} of 41.8 G and one ^{31}P (A_P) of 115 G and 30 G linewidth (Lorentzian).

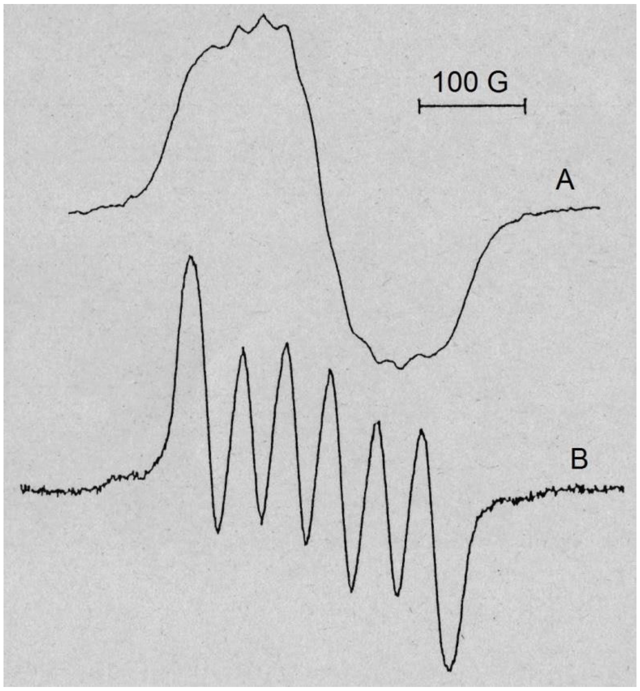

Figure S4. X-band EPR spectrum of the assumed $[\{Re(CO)_3Cl\}_2(\mu\text{-adcpip})]^{\bullet-}$ in toluene before (**A**) and after (**B**) addition of a small amount of MeCN, measured at 9.864 GHz and 4.0 modulation amplitude; simulation of (**A**), see Figure S1; simulation of (**B**) (not shown): A_{Re} = 42.7 G, linewidth = 30 G and Lorentzian lines.

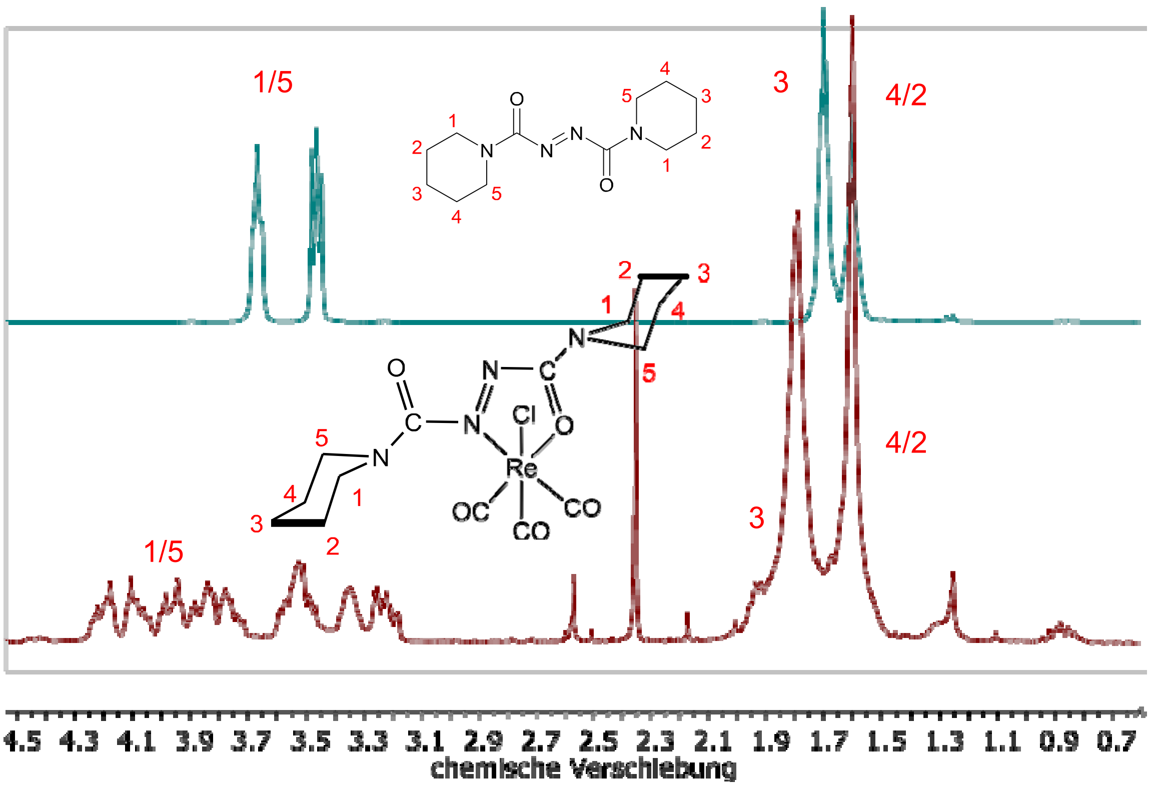

Figure S5. 300 MHz ^{1}H NMR spectra of adcpip (top) and [Re(CO)$_3$Cl(adcpip)] (bottom) in $CDCl_3$.

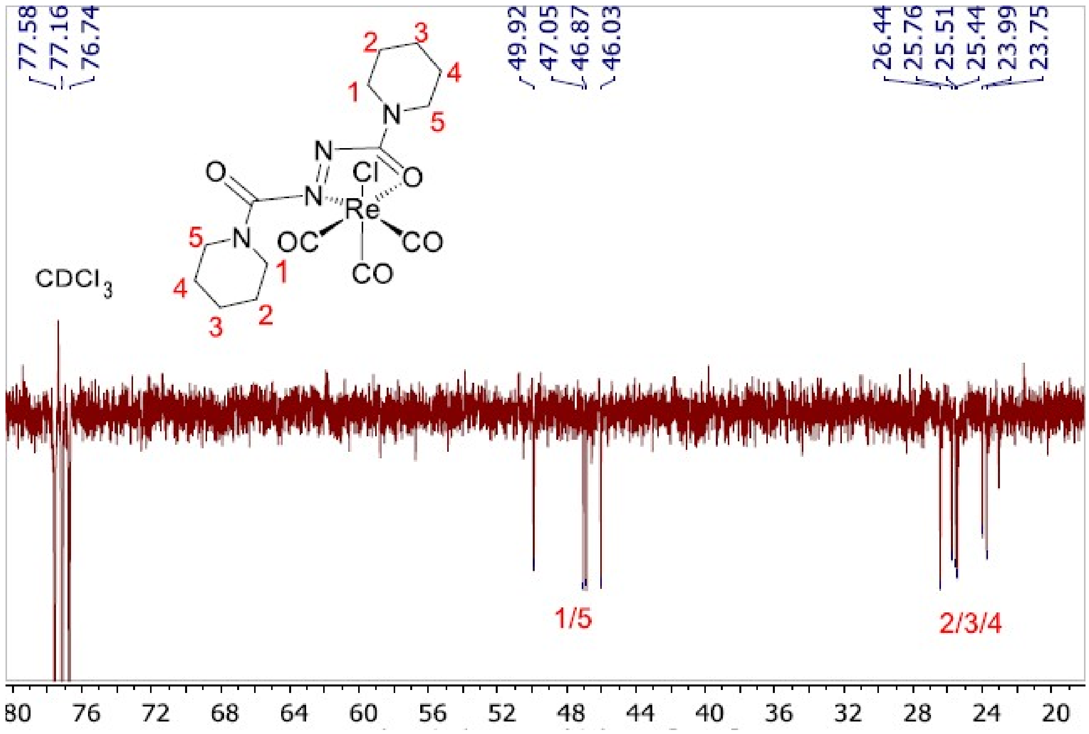

Figure S6. 75 MHz ^{13}C DEPTQ NMR spectrum of [Re(CO)$_3$Cl(adcpip)] in $CDCl_3$.

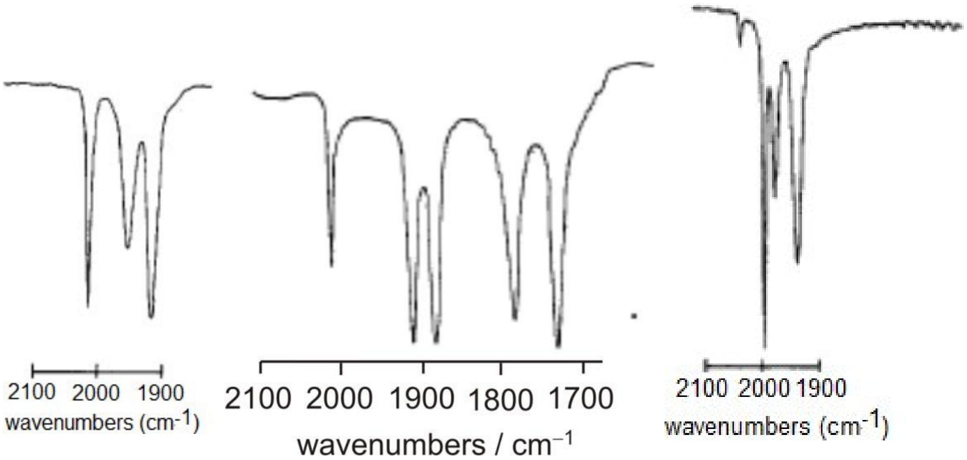

Figure S7. IR spectra of [Re(CO)$_3$Cl(adcpip)] (left), [Re(CO)$_3$(PPh$_3$)(adcpip)]Cl (middle), and [Re(CO)$_4$(μ-Cl)$_2$Re(CO)$_4$] (right) in CH_2Cl_2 solution.

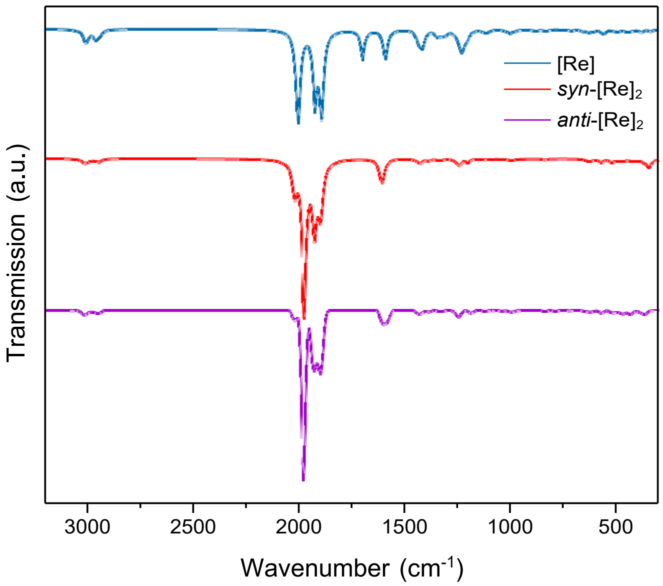

Figure S8. DFT-calculated IR spectra of [Re(CO)$_3$Cl(adcpip)] [Re], and [{Re(CO)$_3$Cl}$_2$(μ-adcpip)] (*anti*-[Re]$_2$, and *syn*-[Re]$_2$) ; TPSSh(def2-TZVP(+def2-ECP for Re)/(CPCMC(THF) level of theory.

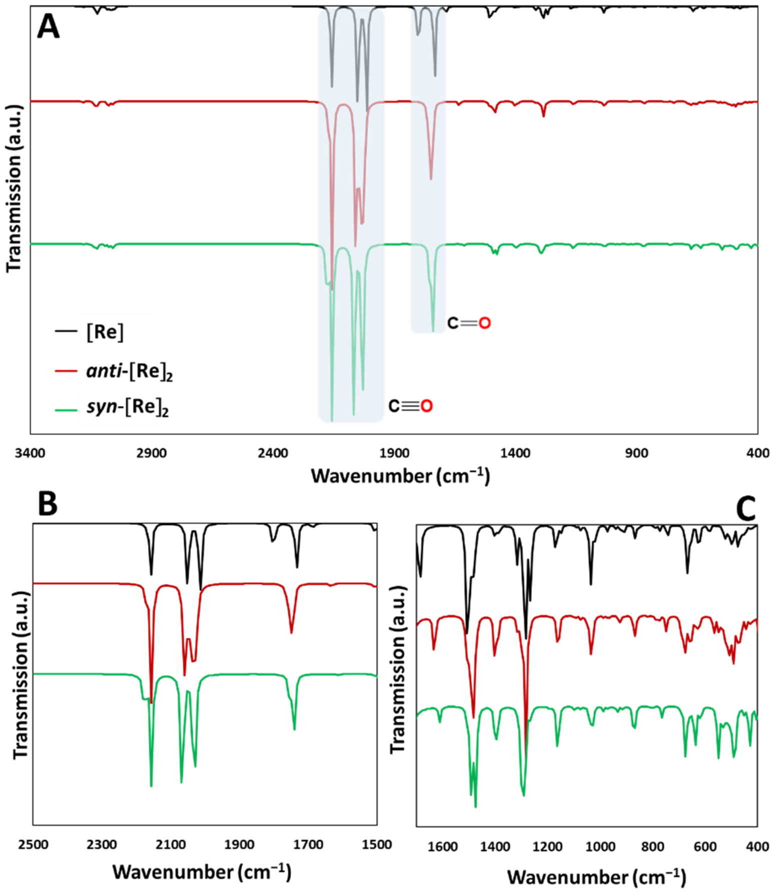

Figure S9. (**A**) DFT calculated IR spectra of [Re(CO)$_3$Cl(adcpip)] [Re], and [{Re(CO)$_3$Cl}$_2$(μ-adcpip)] (*anti*-[Re]$_2$ and *syn*-[Re]$_2$), solvent: THF, functional: M06-2X, basis set: def2TZVP for C, H, N, O, Cl and LANL2DZ for Re; (**B**,**C**) Magnified view (400-1700 cm^{-1} and 1500-2500 cm^{-1}) of the IR spectra of [Re], and *anti*-[Re]$_2$, and *syn*-[Re]$_2$.

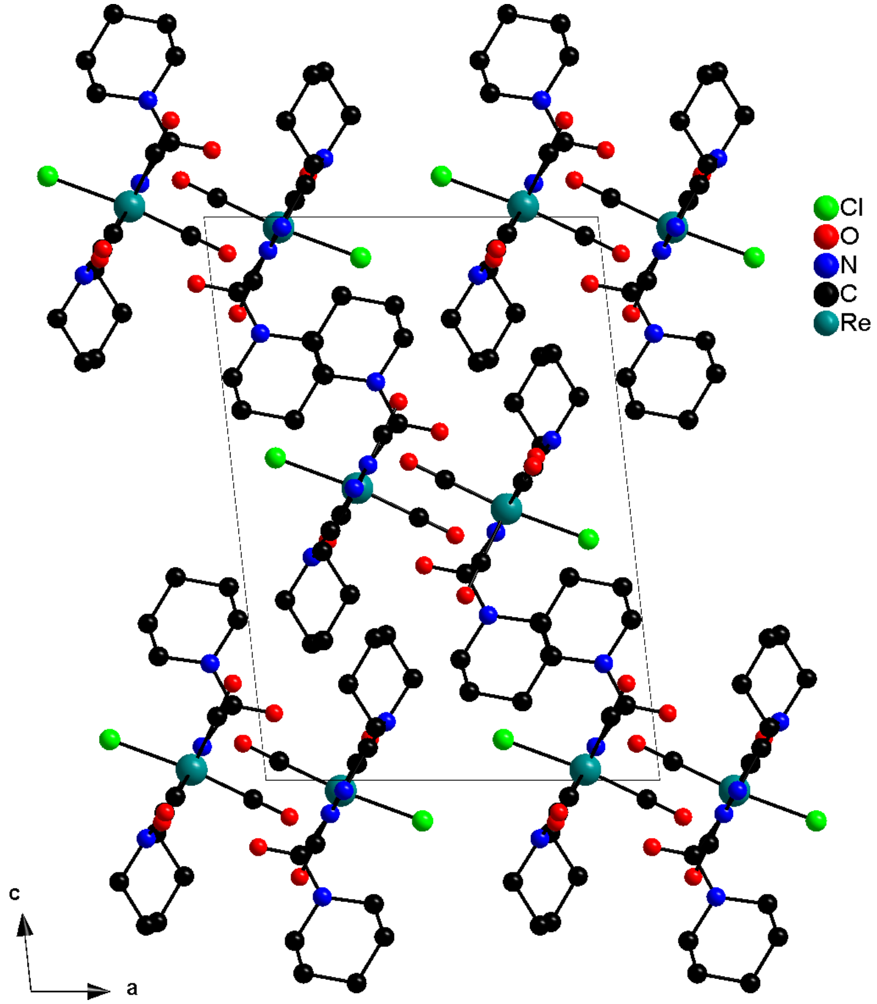

Figure S10. View on the crystal structure of [Re(CO)$_3$Cl(adcpip)] along the crystallographic *b* axis.

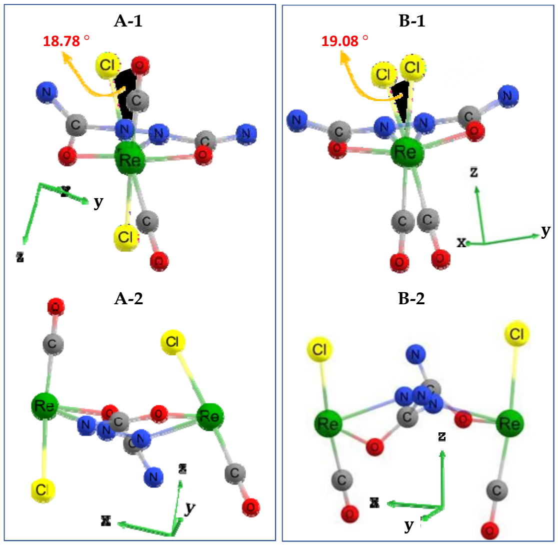

Figure S11. Views on the optimised structures in the *S$_0$* ground state for [{Re(CO)$_3$Cl}$_2$(μ-adcpip)] A: in *anti* configuration (*anti*-[Re]$_2$, and B: in *syn* configuration (*syn*-[Re]$_2$); M06-2X/def2TZVP/LANL2DZ/CPCM(THF) level of theory.

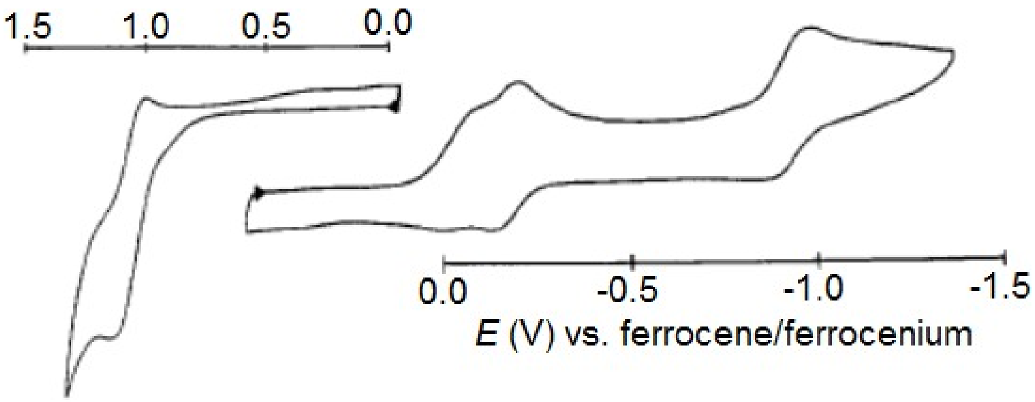

Figure S12. Cyclic voltammogramms of [{Re(CO)$_3$Cl}$_2$(adc-OEt)] in 0.1 M *n*-Bu$_4$NPF$_6$/DCE.

23

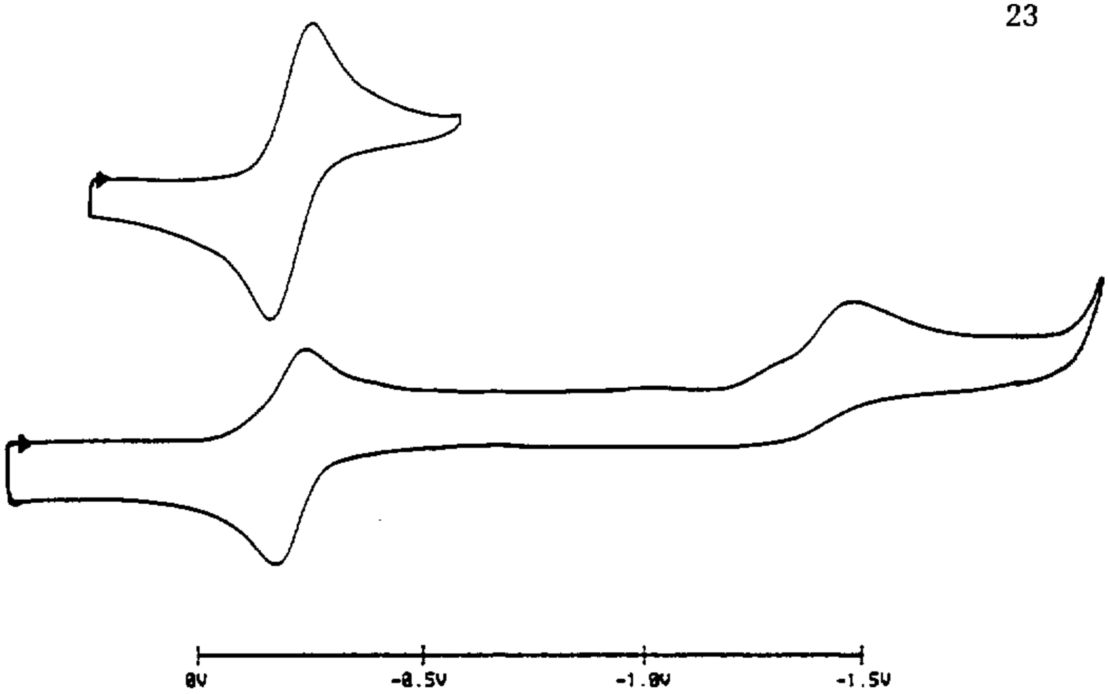

Figure S13. Cyclic voltammogramm of [{Re(CO)$_3$Cl}$_2$(μ-adcpip)] in *n*-BuNPF$_6$/DCE.

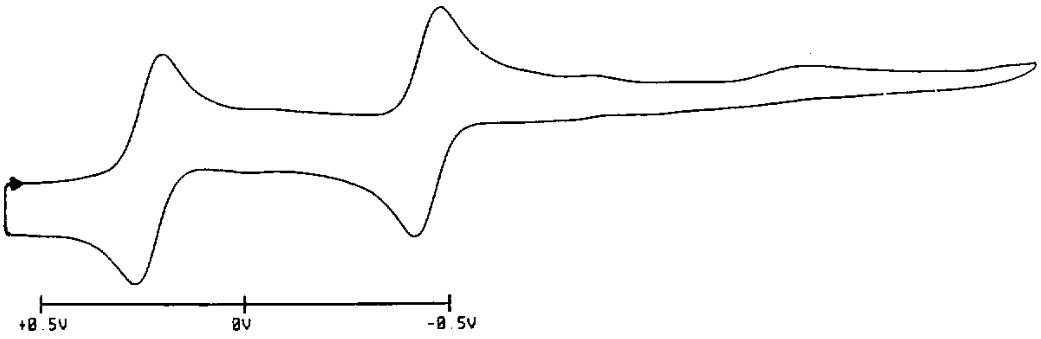

Figure S14. Cyclic voltammogramm of [Re(CO)$_3$(PPh$_3$)(adcpip)]Cl in *n*-BuNPF$_6$/DCE.

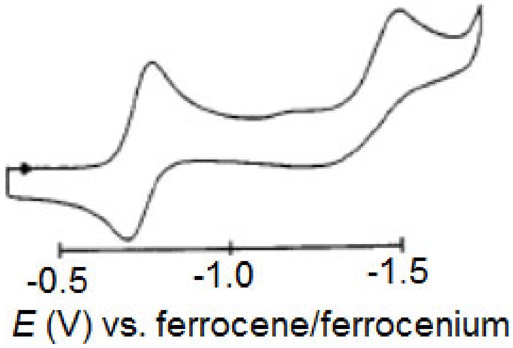

Figure S15. Cyclic voltammogramms of $[Re(CO)_3Cl(pacOEt)]$ in 0.1 M *n*-Bu_4NPF_6/DCE.

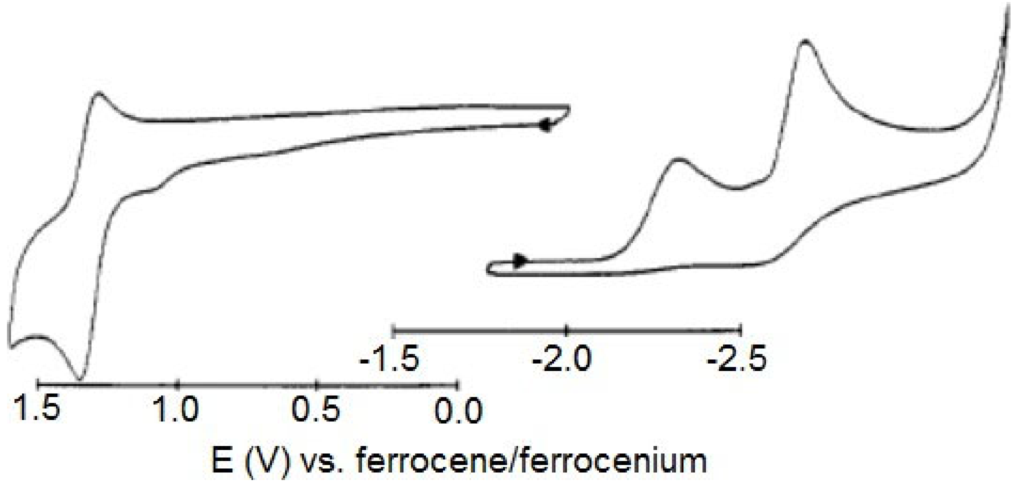

Figure S16. Cyclic voltammogramms of $[Re_2(\mu\text{-}Cl)_2(CO)_8]$ in 0.1 M *n*-Bu_4NPF_6/DCE.

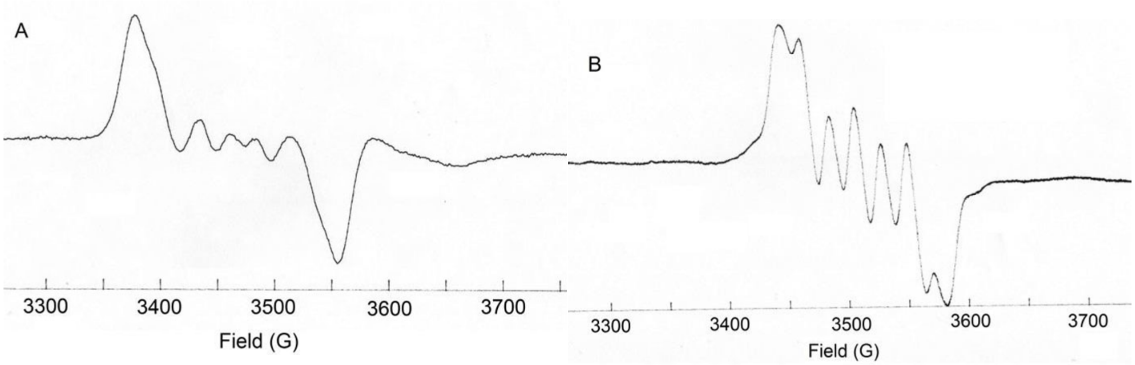

Figure S17. X-band EPR spectrum of the assumed $[Re(CO)_3(CH_2Cl_2)(adcpip)]^{\bullet}$ (A) generated from $[\{Re(CO)_3Cl\}_2(\mu\text{-adcpip})]$ and $CoCp_2$ in CH_2Cl_2 in solution at 298 K. Simulation using using A_{Re} = 22 G, linewidth = 15 G and Lorentzian lines (not shown). (B) EPR spectrum of the assumed $[Re(CO_3)(NEt_3)(adcpip)]^{\bullet}$ in CH_2Cl_2 at 298 K, obtained from $[\{Re(CO)_3Cl\}_2(\mu\text{-adcpip})]$ after reaction with a small amount of NEt_3. Simulation using using A_{Re} = 23.3 G, linewidth = 15 G and Lorentzian lines (not shown).

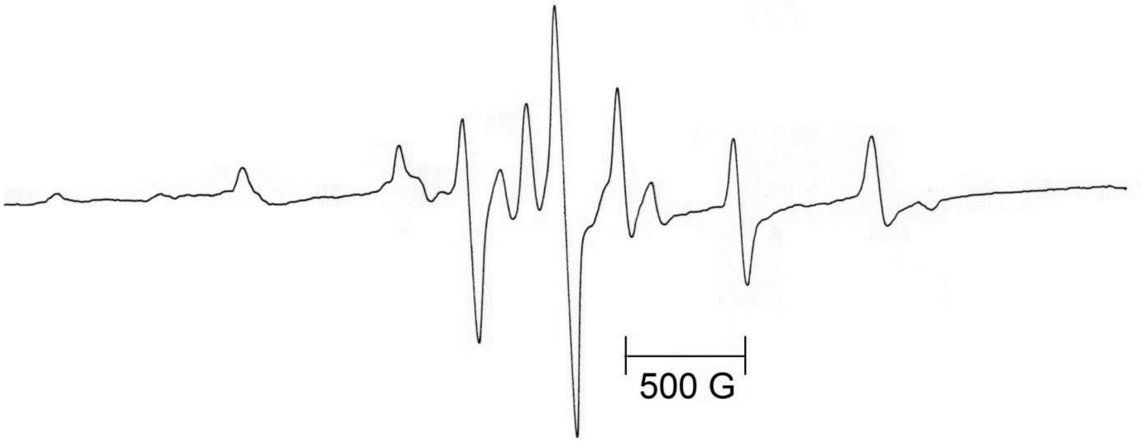

Figure S18. X-band EPR spectra of the assumed $[Re(CO)_3Cl(adcpip)]^{\bullet-}$ in glassy frozen acetone matrix at 4 K.

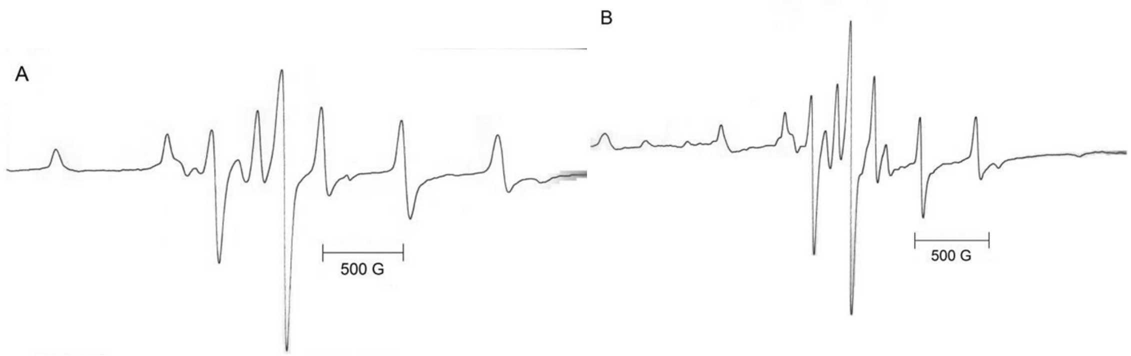

Figure S19. X-band EPR spectra of the assumed $[Re(CO)_3Cl(adcOEt)]^{\bullet-}$ (A) and $[Re(CO)_3Cl(adcO\textit{i}Pr)]^{\bullet-}$ (B) in glassy frozen acetone matrix 4 K.

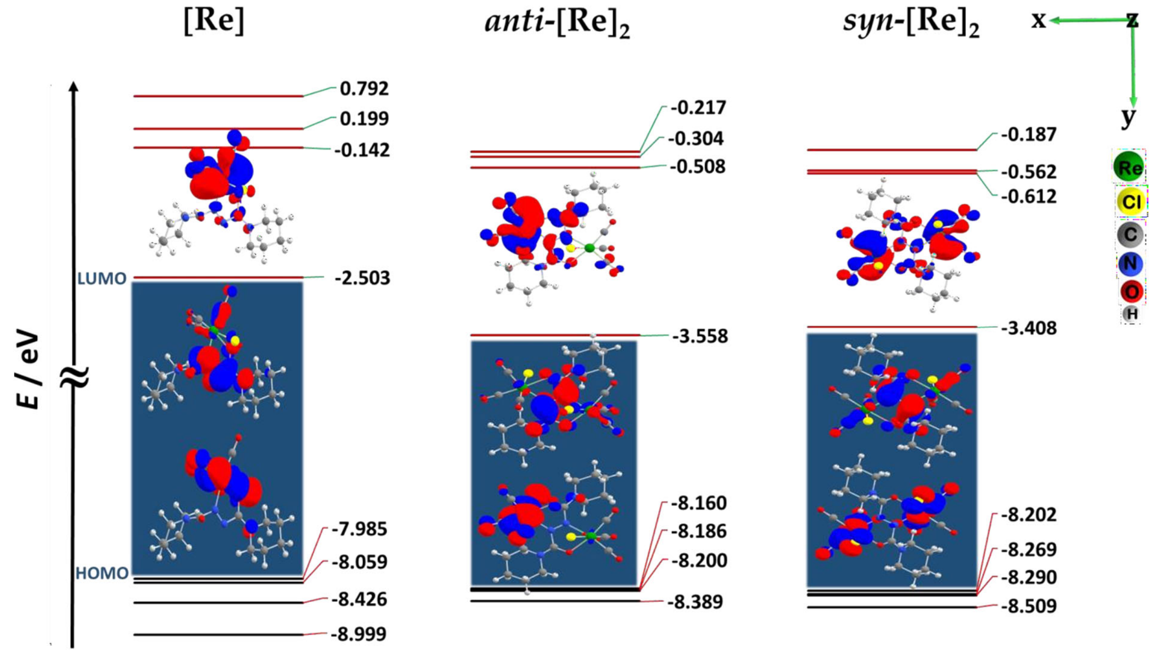

Figure S20. DFT-calculated energies of occupied MOs (blue) and unoccupied MOs (red) for the Re complexes [Re], *anti*-$[Re]_2$ and *syn*-$[Re]_2$; M06-2X/def2TZVP/LANL2DZ for Re/CPCM(THF) level of theory.

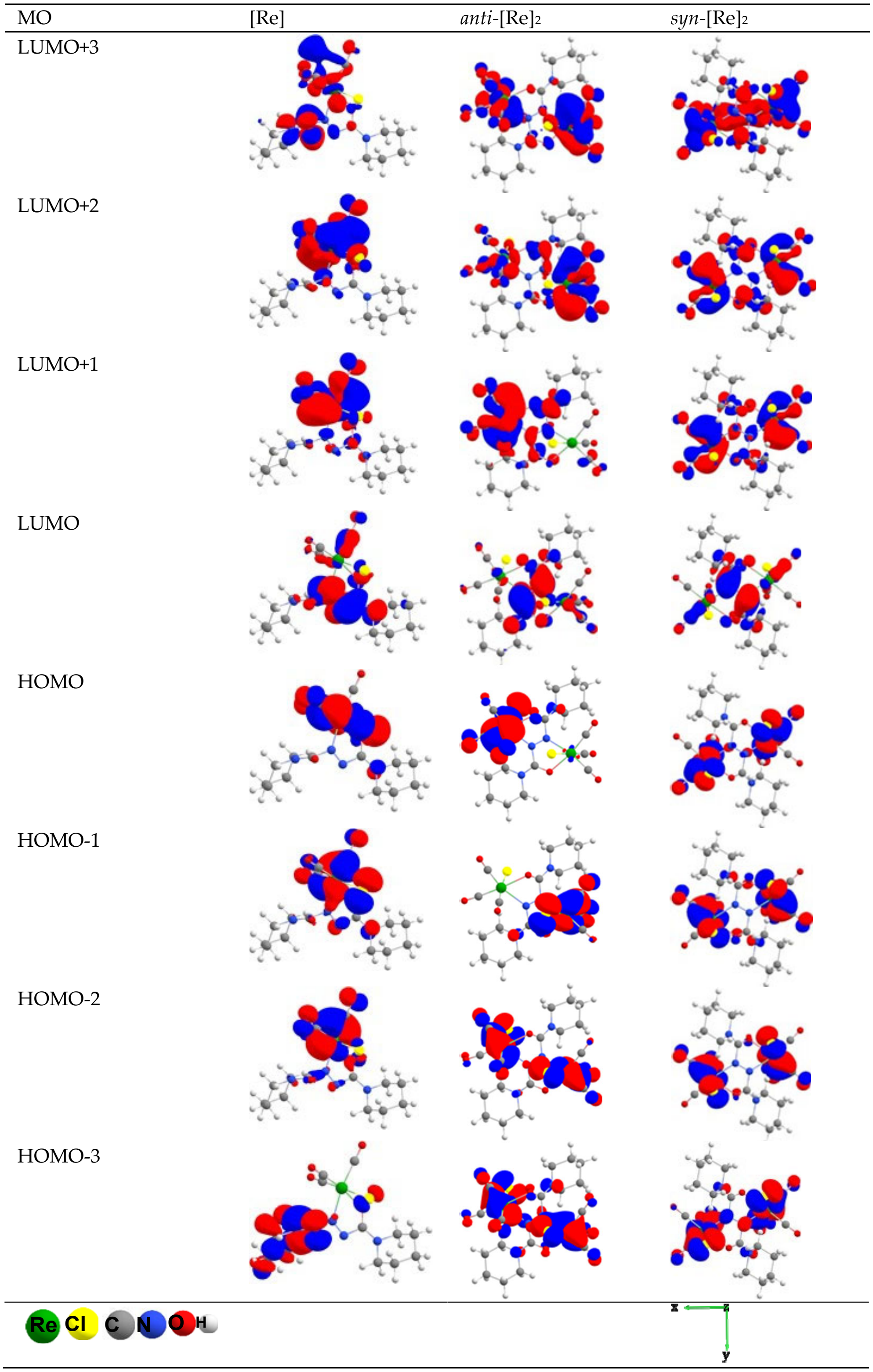

Figure S21. Frontier orbital landscape in the ground state (S_0) for [Re(CO)$_3$Cl(adcpip)] [Re], and [{Re(CO)$_3$Cl}$_2$(μ-adcpip)] (*anti*-[Re]$_2$ and *syn*-[Re]$_2$); M062X/def2TZVP/LANL2DZ for Re/CPCM(THF) level of theory.

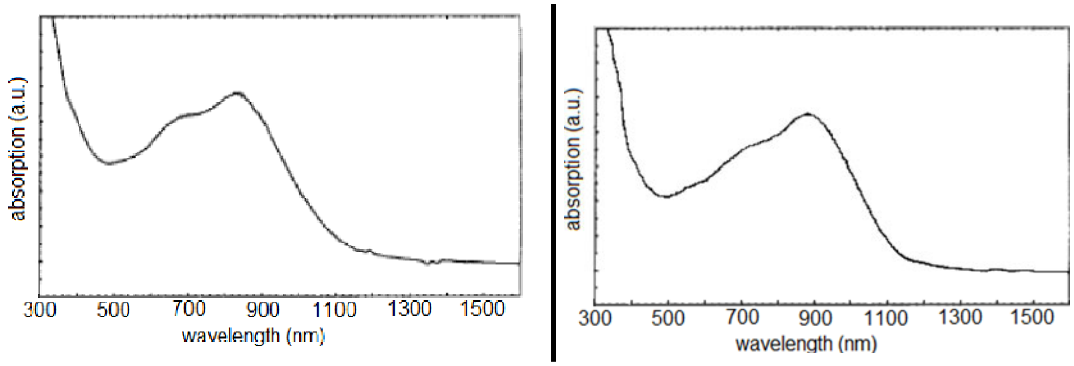

Figure S22. UV-vis-NIR absorption spectrum of [{Re(CO)$_3$Cl}$_2$(O*i*Pr)] (left) and [{Re(CO)$_3$Cl}$_2$(μ-adcOEt)] (right) in CH_2Cl_2.

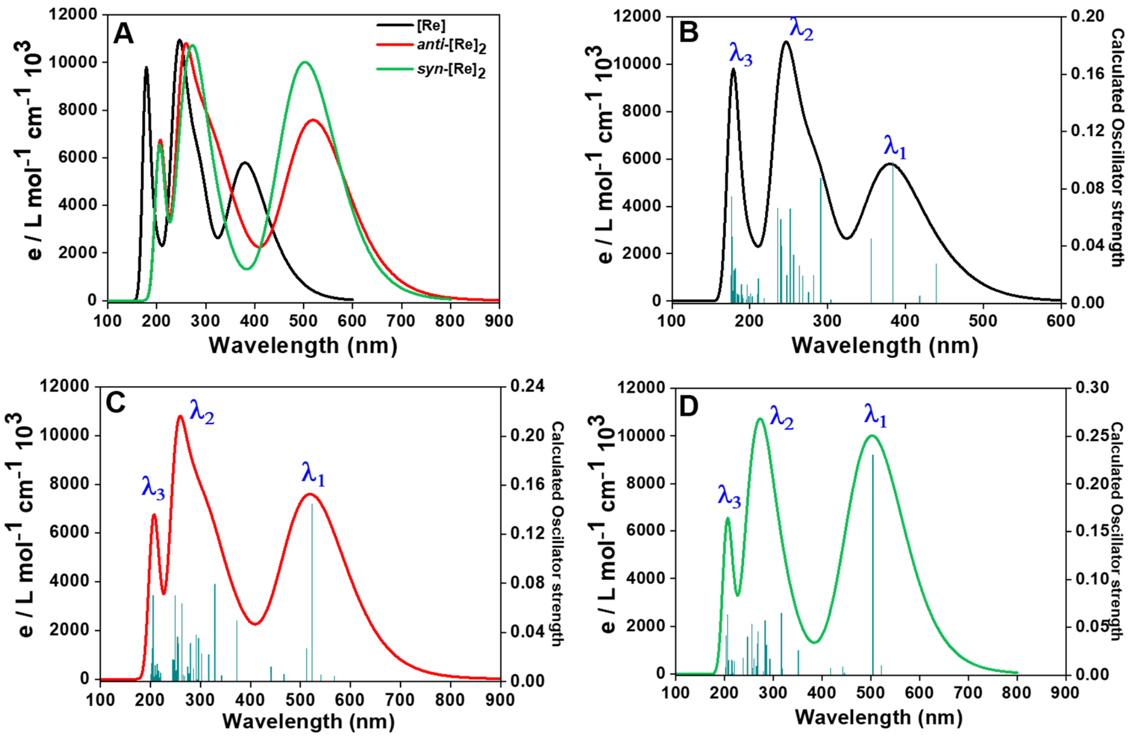

Figure S23. TD-DFT-calculated UV-vis absorption spectra **A:** Overlay spectra of [Re], *anti*-[Re]$_2$, and *syn*-[Re]$_2$; **B:** [Re]; **C:** *anti*-[Re]$_2$; **D:** *syn*-[Re]$_2$; M06-2X/def2-TZVP/LANL2DZ for Re/CPCM(THF) level of theory.

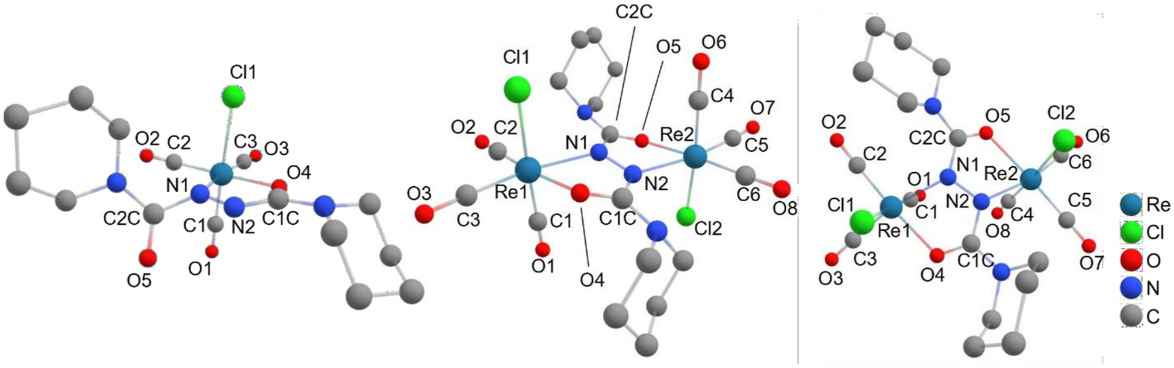

Figure S24. DFT-optimised structures in the D_0 ground state for [Re]$^{\bullet-}$, *anti*-[Re]$_2^{\bullet-}$, and *syn*-[Re]$_2^{\bullet-}$; H atoms omitted for clarity; BP86/def2-TZVP(+def2-ECP for Re)/CPCMC(THF) level of theory.

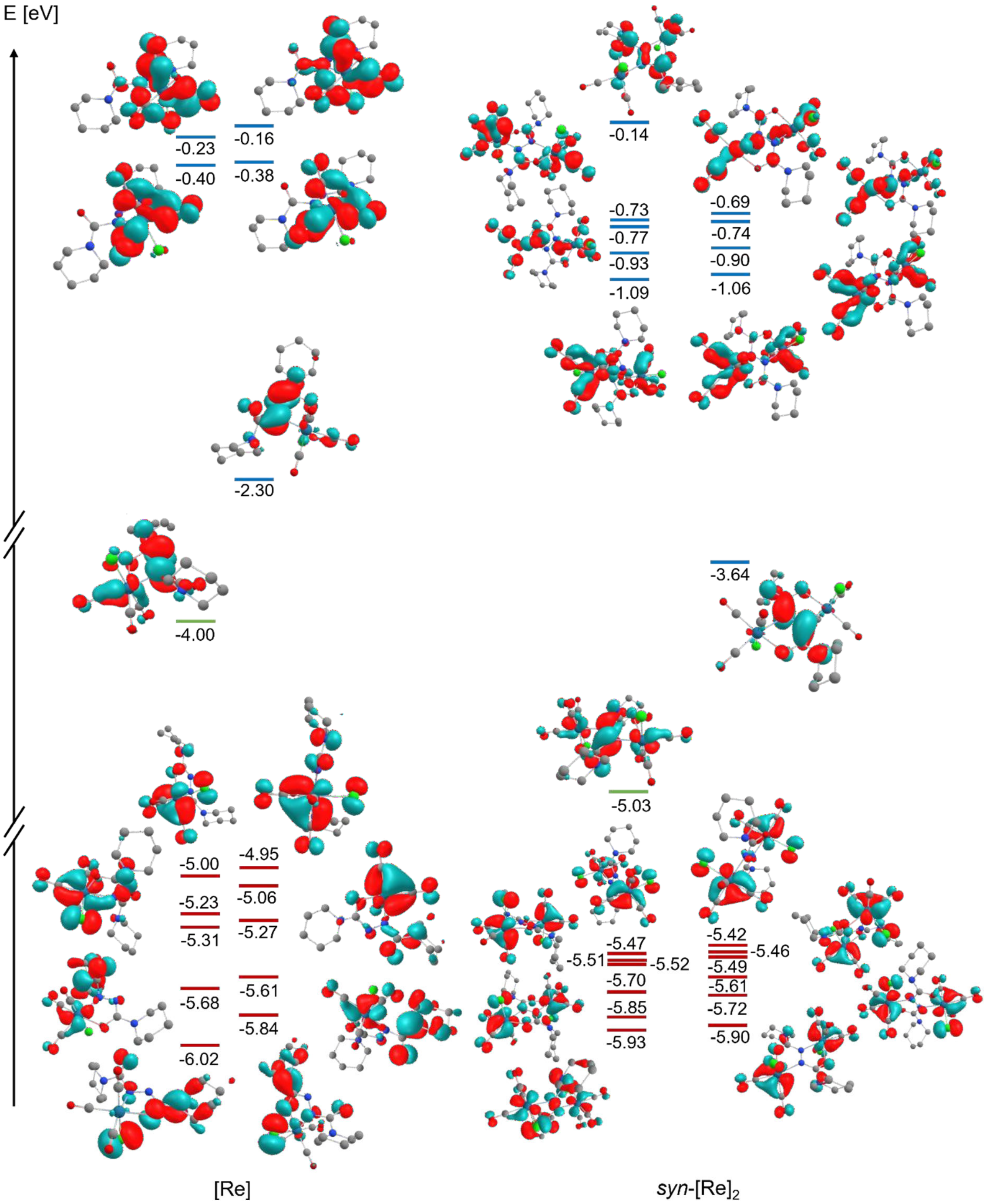

Figure S25. DFT-calculated frontier orbital landscape in the ground state (D_0) for $[Re]^{\bullet-}$ and *anti*-$[Re]_2^{\bullet-}$; TPSSh/def2-TZVP(+def2-ECP for Re)/CPCMC(THF) level of theory.

Supporting Tables

Table S1. Crystal Structure and solution data of [Re(CO)$_3$Cl(adcpip)].

		[Re(CO)$_3$Cl(adcpip)]
Formula / Formula weigth (g/mol)		$C_{15}H_{20}ClN_4O_5Re$ / 558.00
Temperature (K) / Wavelength		100(2) / Mo K$_\alpha$ (λ = 0.71073 Å)
Crystal system / Space group		monoclinic / $P2_1/n$
Unit cell	a (Å)	11.0824(7)
	b (Å)	10.8130(7)
	c (Å)	15.9619(10)
	β (°)	96.288(2)
Volume (Å^3) / Z		1901.3(2) / 4
Calculated density (g cm^{-1})		1.949
Absorption coefficient (cm^{-1})		6.57
F(000)		1080
Crystal colour/shape		purple needle
Measurement device type		*Bruker D8 Venture*
Theta range for data collection (°)		2.3 to 33.1
Index ranges		–11 ≤ h ≤ 11, –26 ≤ k ≤ 25, –27 ≤ l ≤ 27
Reflections collected		144888
Independent reflections	total/I > 2σ(I)	7262/5642
Completeness to theta		100%
Data / restraints / parameters		7262/0/235
R_{int}		0.1032
R_σ		0.0315
Goodness-of-fit on F^2		1.10
R_1/wR_2 (I > 2σ(I))		0.0226/0.0508
R_1/wR_2 (all data)		0.0307/0.0508
Largest diff. peak and hole (e Å^{-1})		1.19/–2.33
CCDC		2194078

Table S2. Selected metrics from the crystal structure of [Re(CO)$_3$Cl(adcpip)].

**Bond lengths (Å)**			**Angles (°)**	
Re1–Cl1	2.457(1)		Cl1–Re1–C1	174.5(1)
Re1–C1	1.924(2)		Cl1–Re1–C2	95.6(1)
Re1–C2	1.897(2)		Cl1–Re1–C3	87.0(1)
Re1–C3	1.942(2)		Cl1–Re1–O4	81.6(1)
Re1–O4	2.151(1)		Cl1–Re1–N1	89.6(1)
Re1–N1	2.117(2)		O4–Re1–N1	71.6(1)
N1–N2	1.261(2)		O4–Re1–C1	95.2(1)
C1C–O4	1.257(3)		O4–Re1–C2	170.2(1)
C2C–O5	1.211(3)		O4–Re1–C3	100.9(1)
N1–C2C	1.489(2)		C1–Re1–C2	88.3(1)
N2–C1C	1.443(2)		C1–Re1–C3	89.2(1)
C1–O1	1.148(3)		C1–Re1–N1	93.7(1)
C2–O2	1.156(3)		C2–Re1–C3	88.3(1)
C3–O3	1.145(3)		C2–Re1–N1	99.0(1)
C1C–N3	1.317(3)		C3–Re1–N1	172.2(1)
C2C–N4	1.330(3)		N2–N1–Re1	123.1(1)
			N4–C2C–N1	115.1(2)

			N3–C1C–N2	115.1(2)
Dihedral angles (°)				
Re1–O4–C1C–N2	3.3(2)		N1–N2–C1C–N3	176.5(2)
Re1–N1–N2–C1C	0.9(2)		N2–N1–C2C–N4	99.8(2)
C2C–N1–N2–C1C	172.9(2)			

Table S3A. Selected DFT-calculated metrics of [Re], *anti*-$[Re]_2$ and *syn*-$[Re]_2$, in comparison with those of $[Re]^{\bullet-}$, *anti*-$[Re]_2^{\bullet-}$ and *syn*-$[Re]_2^{\bullet-}$; at BP86/def2-TZVP(+def2-ECP for Re)/CPCMC(THF) level of theory.

**Bond length (Å)**	**[Re]**	**$[Re]^{\bullet-}$**	**Δ[a]**	***anti*-[Re]**	***anti*-$[Re]_2^{\bullet-}$**	**Δ[a]**	***syn*-$[Re]_2$**	***syn*-$[Re]_2^{\bullet-}$**	**Δ[a]**
Re1–Cl1	2.473	2.532	+0.059	2.456	2.532	+0.076	2.446	2.522	+0.076
Re1–C1	1.933	1.906	–0.027	1.937	1.912	–0.025	1.937	1.915	–0.022
Re1–C2	1.912	1.909	–0.003	1.911	1.903	–0.008	1.907	1.898	–0.009
Re1–C3	1.969	1.932	–0.037	1.947	1.911	–0.036	1.951	1.916	–0.035
Re1–O4	2.169	2.149	–0.020	2.183	2.230	+0.047	2.191	2.271	+0.080
Re1–N1	2.078	2.159	+0.081	2.145	2.322	+0.177	2.131	2.273	+0.142
Re2–Cl2	-	-	-	2.466	2.532	+0.066	2.449	2.524	+0.075
Re2–C4	-	-	-	1.932	1.912	–0.020	1.936	1.915	–0.021
Re2–C5	-	-	-	1.911	1.903	–0.008	1.907	1.897	–0.010
Re2–C6	-	-	-	1.949	1.911	–0.038	1.950	1.918	–0.032
Re2–O5	-	-	-	2.162	2.230	+0.068	2.202	2.282	+0.080
Re2–N2	-	-	-	2.135	2.321	+0.186	2.134	2.262	+0.128
N1–N2	1.283	1.344	+0.061	1.325	1.362	+0.037	1.321	1.355	+0.034
C1C–O4	1.275	1.293	+0.018	1.268	1.276	+0.008	1.266	1.273	+0.007
C2C–O5	1.227	1.239	+0.012	1.266	1.276	+0.010	1.266	1.272	+0.006
N1–C2C	1.484	1.432	–0.052	1.421	1.388	–0.033	1.434	1.395	–0.039
N2–C1C	1.414	1.360	–0.054	1.428	1.388	–0.040	1.428	1.394	–0.034
C1≡O1	1.164	1.174	+0.010	1.161	1.170	+0.009	1.163	1.169	+0.006
C2≡O2	1.167	1.174	+0.007	1.165	1.171	+0.006	1.166	1.172	+0.006
C3≡O3	1.157	1.170	+0.013	1.157	1.168	+0.011	1.157	1.168	+0.011
C4≡O6	-	-	-	1.166	1.170	+0.004	1.163	1.169	+0.006
C5≡O7	-	-	-	1.166	1.171	+0.005	1.166	1.171	+0.005
C6≡O8	-	-	-	1.157	1.168	+0.011	1.157	1.168	+0.011
Re1···Re2	-	-	-	4.812	5.354	+0.542	4.766	4.982	+0.216
Angles (°)									
Cl1–Re1–C1	177.4	177.7	+0.3	179.6	174.7	–4.9	177.7	175.8	–1.9
Cl1–Re1–C2	93.6	94.1	+0.5	94.2	97.4	+3.2	95.2	96.3	+1.1
Cl1–Re1–C3	88.6	91.4	+2.8	93.1	90.9	–2.2	92.9	93.6	+0.7
Cl1–Re1–O4	81.9	82.3	+0.4	82.5	82.4	–0.1	82.7	82.2	–0.5
Cl1–Re1–N1	90.0	86.9	–3.1	89.2	90.6	+1.4	90.5	86.6	–3.9
O4–Re1–N1	72.1	73.0	+0.9	73.1	69.2	–3.9	73.3	70.7	–2.6
O4–Re1–C1	96.9	95.4	–1.5	97.5	92.3	–5.2	95.0	94.2	–0.8
O4–Re1–C2	169.4	172.4	+3.0	176.2	173.3	–2.9	175.8	174.8	–1.0
O4–Re1–C3	98.5	97.5	–1.0	93.1	99.4	+6.3	95.1	97.7	+2.6
C1–Re1–C2	87.9	88.2	+0.3	85.8	87.9	+2.1	87.0	87.1	+0.1
C1–Re1–C3	91.0	89.4	–1.6	87.3	89.6	+2.3	87.7	89.1	+1.4
C1–Re1–N1	91.8	92.0	+0.2	90.4	87.8	–2.6	88.5	90.1	+1.6
C2–Re1–C3	91.0	89.2	–1.8	89.0	87.3	–1.7	88.6	87.4	–1.2
C2–Re1–N1	98.3	100.3	+2.0	105.0	104.1	–0.9	103.2	104.3	+1.1
C3–Re1–N1	170.7	170.5	–0.2	165.5	168.2	+2.7	167.3	168.2	+0.9
Cl2–Re2–C4	-	-	-	176.3	174.7	–1.6	177.7	176.0	–1.7
Cl2–Re2–C5	-	-	-	90.7	97.4	+6.7	94.6	95.0	+0.4
Cl2–Re2–C6	-	-	-	87.2	90.9	+3.7	93.3	93.9	+0.6
Cl2–Re2–O5	-	-	-	85.4	82.4	–3.0	82.3	81.6	–0.7
Cl2–Re2–N2	-	-	-	84.6	89.9	+5.3	89.5	86.0	–3.5
O5–Re2–N2	-	-	-	72.3	69.2	–3.1	73.2	71.0	–2.2
O5–Re2–C4	-	-	-	94.7	92.3	–2.4	95.5	95.3	–0.2

O5–Re2–C5	-	-	-	172.0	173.3	+1.3	174.4	173.2	–1.2
O5–Re2–C6	-	-	-	97.6	99.4	+1.8	95.3	98.0	+2.7
C4–Re2–C5	-	-	-	89.7	87.9	–1.8	87.5	87.8	+0.3
C4–Re2–C6	-	-	-	89.1	89.6	+0.5	87.7	89.0	+1.3
C4–Re2–N2	-	-	-	99.0	88.5	–10.5	89.0	90.7	+1.7
C5–Re2–C6	-	-	-	89.1	87.3	–1.8	89.5	88.1	–1.4
C5–Re2–N2	-	-	-	100.4	104.1	+3.7	102.1	103.0	+0.9
C6–Re2–N2	-	-	-	167.5	168.4	+0.9	167.8	168.9	+1.1
N2–N1–Re1	122.7	118.2	-4.5	116.8	114.5	–2.3	117.4	115.8	–1.6
N1–N2–Re2	-	-	-	117.6	114.5	–3.1	117.2	116.2	–1.0
N–C2C–N1	114.7	117.6	+2.9	118.7	119.3	+0.6	117.7	117.2	–0.5
N–C1C–N2	116.4	114.7	–1.7	118.0	119.3	+1.3	118.1	117.4	–0.7
Dihedral angles (°)									
Re1–O1–C–N2	15.4	7.7	–7.7	39.3	27.4	–11.9	–30.4	–17.0	+13.4
Re1–N1–N2–C	–2.9	3.3	+6.2	14.3	–13.8	–28.1	–20.9	–22.5	–1.6
Re2–O2–C–N1	-	-	-	10.8	–27.3	–38.1	–37.2	–23.0	+14.2
Re2–N2–N1–C	-	-	-	25.8	13.8	–12.0	–20.2	–19.6	+0.6
C2C–N1–N2–C1C	168.2	162.9	–5.3	175.3	–179.9	+4.8	–172.9	–172.2	+0.7
Re1–N1–N2–Re2	-	-	-	–135.2	–180.0	–44.8	131.9	130.0	–1.9

[a] Δ = differences between neutral and reduced species.

Table S3B. Selected DFT-calculated metrics of [Re], *anti*-$[Re]_2$ and *syn*-$[Re]_2$; M06-2X/ def2TZVP/LANL2DZ for Re/CPCM(THF) level of theory.

**Bond length (Å)**	**[Re]**	***anti*-$[Re]_2$**	***syn*-$[Re]_2$**		**Angles (°)**	**[Re]**	***anti*-$[Re]_2$**	***syn*-$[Re]_2$**
Re1–Cl1	2.539	2.526	2.522		Cl1–Re1–N1	81.983	80.188	83.154
Re2–Cl2	–	2.525	2.522		Cl2–Re2–N2	–	78.171	83.153
Re1–C3	1.897	1.907	1.906		Cl1–Re1–O1	79.499	80.359	80.362
Re1–C4	1.891	1.885	1.884		Cl2–Re2–O2	–	81.543	80.362
Re1–C5	1.923	1.884	1.915		Cl1–Re1–C3	175.908	172.452	173.324
Re2–C6	–	1.904	1.906		Cl2–Re2–C6	–	176.965	173.324
Re2–C7	–	1.891	1.884		C4–Re1–O1	169.238	173.390	174.498
Re2–C8	–	1.915	1.915		C7–Re2–O2	–	169.494	174.500
Re1–N1	2.197	2.442	2.249		C5–Re1–N1	169.894	162.962	168.071
Re2–N2	–	2.242	2.249		C8–Re2–N2	–	167.167	168.070
Re1–O1	2.239	2.260	2.286		O1–Re1–N1	69.081	68.862	69.953
Re2–O2	–	2.210	2.286		O2–Re2–N2	–	68.183	69.953
N1–N2	1.228	1.236	1.239		Re1–O1–C1	112.957	109.339	107.443
N1–C2	1.481	1.478	1.474		Re2–O2–C2	–	119.220	107.442
N2–C1	1.460	1.473	1.474		Re1–N1–N2	122.283	111.329	117.331
N3–C1	1.311	1.306	1.304		Re2–N2–N1	–	121.172	117.329
N4–C2	1.326	1.311	1.304		C1–N2–N1	110.349	113.939	111.219
C1–O1	1.242	1.232	1.236		O1–C1–N2	119.318	115.689	115.251
C2–O2	1.207	1.235	1.236		C2–N1–N2	113.948	109.719	111.220
C3–O3	1.145	1.141	1.141		N3–C1–N2	114.861	115.985	116.551
C4–O4	1.143	1.143	1.144		N4–C2–N1	114.724	117.337	116.550
C5–O5	1.138	1.140	1.136		O2–C2–N1	115.841	117.816	115.250
C6–O6	–	1.143	1.141		O1–C1–N3	125.766	128.188	128.196
C7–O7	–	1.143	1.144		O2–C2–N4	129.357	124.833	128.196
C8–O8	–	1.137	1.136					
Re1...Re2	–	5.105	5.011					
Dihedral angles (°)								
O1-C1-N2-N1	–15.944	47.009	45.311					
O2–C2–N1–N2	88.043	17.144	45.314		Re1–N1–N2–C1	–4.930	–14.629	–15.639

Re1–O1–C1–N2	27.403	–52.060	–47.968		Re2–N2–N1–C2	–	–22.795	–15.638
Re2–O2–C2–N1	–	–3.886	–47.971		Re1–N1–N2–Re2	–	137.429	141.065

Table S4. Experimental IR data of adc ligands and Re complexes.[a]

**Compound**	υ_{CO}	**Re(CO)**				υ_{CO}	**ligand**
$[Re(CO)_5Cl]$	2050	1983					
$[Re_2(\mu\text{-}Cl)_2(CO)_8]$	2143	1998	1920	1885			
adcpip						1704	
$[Re(CO_3)Cl(adcpip)]$	2040	1960	1920			1725	1640
$[\{Re(CO_3)Cl\}_2(\mu\text{-}adcpip)]$	2015	1913				1590	
pacOEt						1788	
$[Re(CO_3)Cl(pacOEt)]$	2022	1962	1928			1482	
adcOEt						1778	
$[\{Re(CO_3)Cl\}_2(\mu\text{-}adcOEt)]$	2019	1916				1667	
adcO*i*Pr						1773	
$[\{Re(CO_3)Cl\}_2(\mu\text{-}adcO\textit{i}Pr)]$	2021	1919				1665	
adcOBzl						1756	
adcO*t*Bu						1768	

[a] Measured in CH_2Cl_2 or DCE solution.

Table S5. Electrochemical data of adc ligands.[a]

**Compound**	$E_{1/2}$ **Red1**	E_{pc} **Red2**	ΔE **Red1-Red2**
adcpip	–1.52	–2.25	0.73
adcO*t*Bu	–1.20	–1.84	0.64
adcOEt	–1.02	–1.65	0.63
adcO*i*Pr	–1.03	–1.67	0.64
adcOBzl	–0.91	–1.60	0.69
pacOEt	–1.96	–	-

[a] Potentials in V vs ferrocene/ferrocenium, recorded in 0.1 M nBu_4NPF_6/CH_2Cl_2, half-wave potentials $E_{1/2}$ for reversible waves and E_{pc} = cathodic peak potential for irreversible waves, scan rate = 100 mV/s.

Table S6. Selected X-band EPR data of reduced Re complexes.[a.]

**Assumed Compound**	g_{iso}	A_{Re} **/ G**	**ref.**
$[\{Re(CO)_3Cl\}_2(\mu\text{-}adcpip)]^{\bullet-}$	2.0165	22.2	this work
$[Re(CO)_3(CH_2Cl_2)(adcpip)]^{\bullet}$	2.0167	30.8	this work
$[Re(CO)_3(MeCN)(adcpip)]^{\bullet}$	2.0177	42.7	this work
$[Re(CO)_3(NEt_3)(adcpip)]^{\bullet}$	2.0188	23.3	this work
$[Re(CO)_3(PPh_3)(adcpip)]^{\bullet}$ [b]	2.0192	41.8	this work
$[Re(CO)_3Cl(bpy)]^{\bullet-}$	2.0032	12.0	59
$[Re(CO)_3Cl(apy)]^{\bullet-}$	2.0041	23.8	47

[a] Recorded at 298 K. [b] A_P = 115 G.

Table S7. UV-vis long-wavelength absorption maximum of $[\{Re(CO)_3Cl\}_2(\mu\text{-}adcpip)]$.[a]

λ_{max} **/ nm**	ν_{max} **/ cm^{-1}**	**solvent**	E_T	E^*_{MLCT}	ε_r	μ_0	**AN**
869	11510	toluene	33.9	0.30	2.4	0.4	3.3
853	11710	Et_2O	34.6	0.32	4.2	1.25	
839	11910	THF	37.4	0.59	7.4	1.7	8
852	11940	CH_2Cl_2	41.1	0.67	8.9	1.5	20.4
842	11880	DCE	41.9	0.64	10.4	1.75	
827	12090	acetone	42.2	0.82	20.7	2.7	12.5
822	12170	MeCN	46.0	0.90	37.5	3.5	18.9
811	12330	DMSO	45.0	1.00	48.9	3.9	19.3

[a] Measured in THF, absorption maxima λ in nm. E_T = Dimroth-Reichardt parameter in kcal/mol,[1] E^*_{MLCT} = solvent parameter by Manuta and Lees,[2,3] ε_r = relative dielectricity constant at 25°C,[1] μ_0 = dipolar moment in Debye at 25°C,[1] AN = Gutman acceptor number.[4]

Table S8. Selected TD-DFT calculated vertical $S_0 \rightarrow S_n$ transitions for [Re]; TPSSh/def2-TZVP(+def2-ECP for Re)/CPCMC(THF) level of theory.

n	wavelength (nm)	f_{osc}	participating MOs (contribution)
1	846.2	0.00349	HOMO→LUMO (91%), H-1→LUMO (8%)
2	675.8	0.00131	H-2→LUMO (99%)
3	571.7	0.03337	H-3→LUMO (66%), H-1→LUMO (28%)
4	502.9	0.10666	H-1→LUMO (44%), H-3→LUMO (32%), H-5→LUMO (11%)
5	450.5	0.05503	H-4→LUMO (85%), H-5→LUMO (5%)
6	390.0	0.06197	H-5→LUMO (59%), H-6→LUMO (25%), H-8→LUMO (5%)
7	374.0	0.13825	H-6→LUMO (64%), H-5→LUMO (14%), H-7→LUMO (8%), H-4→LUMO (5%)
8	341.8	0.05311	H-7→LUMO (47%), H-8→LUMO (42%)
9	317.3	0.03743	H-8→LUMO (46%), H-7→LUMO (31%)
10	309.3	0.01029	HOMO→L+1 (84%)
16	282.6	0.01250	HOMO→L+2 (57%), H-2→L+1 (17%), H-1→H+2 (14%)
18	270.0	0.01932	H-1→L+2 (30%), HOMO→L+2 (21%), HOMO→L+3 (20%), H-2→L+1 (16%)
21	263.3	0.02261	H-15→LUMO (81%), H-16→LUMO (6%)
22	254.9	0.01078	H-2→L+2 (37%), H-1→L+3 (17%), H-1→L+2 (13%), H-2→L+1 (12%)
25	248.1	0.01884	HOMO→L+4 (50%), HOMO→L+3 (32%)
30	240.7	0.01332	H-3→L+1 (47%), H-1→L+4 (25%), H-2→L+3 (21%)
33	234.8	0.01313	H-3→L+2 (57%), H-1→L+5 (8%), H-1→L+6 (8%), H-1→L+4 (5%)

Table S9. Selected TD-DFT calculated vertical $S_0 \rightarrow S_n$ transitions for *anti*-$[Re]_2$; TPSSh/def2-TZVP(+def2-ECP for Re)/CPCMC(THF) level of theory.

n	wavelength (nm)	f_{osc}	participating MOs (contribution)
1	1219.1	0.00285	H-1→LUMO (98%)
2	1208.3	0.00223	H-2→LUMO (93%)
3	846.9	0.00521	H-4→LUMO (69%), H-3→LUMO (27%)
4	764.7	0.04486	H-5→LUMO (64%), H-3→LUMO (16%), HOMO→LUMO (12%), H-4→LUMO (6%)
5	750.8	0.25761	HOMO→LUMO (62%), H-4→LUMO (14%), H-3→LUMO (9%)
6	597.4	0.13804	H-3→LUMO (38%), H-5→LUMO (29%), H-4→LUMO (9%), HOMO→LUMO (7%)
7	561.7	0.08000	H-6→LUMO (91%)
8	531.0	0.00727	H-7→LUMO (93%)
9	449.7	0.07208	H-8→LUMO (58%), H-9→LUMO (37%)
10	428.5	0.03500	H-10→LUMO (64%), H-9→LUMO (23%), H-8→LUMO (8%)
11	419.3	0.18703	H-9→LUMO (36%), H-10→LUMO (34%), H-8→LUMO (22%)
12	365.7	0.01270	H-12→LUMO (75%), H-11→LUMO (18%)
13	360.5	0.03091	H-11→LUMO (43%), H-12→LUMO (19%), H-14→LUMO (14%), H-13→LUMO (8%)
20	316.8	0.01478	HOMO→L+1 (91%)
21	308.2	0.02848	H-2→L+1 (74%), HOMO→L+2 (8%)
25	302.4	0.01083	H-1→L+1 (37%), H-1→L+2 (17%), HOMO→L+2 (13%), H-20→LUMO (11%), H-2→L+1 (8%)

Table S10. Selected TD-DFT calculated vertical $S_0 \rightarrow S_n$ transitions for *syn*-[Re]$_2$; TPSSh/def2-TZVP(+def2-ECP for Re)/CPCMC(THF) level of theory.

n	wavelength (nm)	f_{osc}	participating MOs (contribution)
1	1194.9	0.00278	H-1→LUMO (98%)
2	1181.3	0.00048	H-2→LUMO (99%)
3	778.0	0.00099	H-4→LUMO (72%), H-3→LUMO (27%)
4	718.8	0.00222	H-5→LUMO (56%), H-3→LUMO (30%), H-4→LUMO (13%)
5	641.4	0.38674	HOMO→LUMO (86%), H-8→LUMO (7%)
6	545.0	0.01656	H-5→LUMO (35%), H-3→LUMO (35%), H-4→LUMO (11%), H-6→LUMO (7%), H-11→LUMO (6%)
7	531.2	0.03270	H-6→LUMO (88%)
8	516.2	0.00585	H-7→LUMO (95%)
9	438.1	0.01918	H-9→LUMO (96%)
10	423.7	0.04709	H-10→LUMO (34%), H-8→LUMO (32%)
11	406.5	0.14651	H-8→LUMO (56%), H-10→LUMO (34%), HOMO→LUMO (5%)
12	360.5	0.04086	H-11→LUMO (68%), H-12→LUMO (10%), H-17→LUMO (9%)
13	361.3	0.02259	H-12→LUMO (87%), H-11→LUMO (7%)
18	321.6	0.01863	HOMO→L+1 (92%)
20	313.5	0.01058	HOMO→L+2 (44%), H-2→L+1 (32%), H-1→L+2 (9%), H-1→L+1 (6%)
22	311.8	0.01151	H-1→L+1 (45%), H-2→L+1 (12%), L-18→LUMO (9%), H-20→LUMO (9%), H-2→L+2 (6%), L-17→LUMO (5%)
23	308.9	0.01668	H-19→LUMO (77%), HOMO→L+2 (9%)
24	307.3	0.01432	HOMO→L+2 (38%), H-2→L+1 (18%), H-19→LUMO (15%), H-1→L+1 (9%), H-1→L+2 (8%)

Table S11. DFT-calculated absorptions and character of calculated transitions for [Re]; M06-2X/def2TZVP/LANL2DZ for Re/CPCM(THF) level of theory.[a]

Excited state	Oscillator Strength	Calculated λ (nm)	Transitions (Major Contribution)	Assignment
$S_0 \rightarrow S_1$	0.028	439.48	H-1→LUMO (23%)	MLCT/XLCT/L'LCT/IL
			H-7→LUMO (22%)	IL/XLCT
			H-5→LUMO (18%)	XLCT/IL/MLCT
			H-2→LUMO (16%)	MLCT/ L'LCT
$S_0 \rightarrow S_3$	0.099	383.68	H-1→LUMO (58%)	MLCT/XLCT/L'LCT/IL
			H-2→LUMO (34%)	MLCT/ L'LCT
$S_0 \rightarrow S_4$	0.045	355.97	H-2→LUMO (48%)	MLCT/ L'LCT
			H-7→LUMO (14%)	IL/XLCT
			H-1→LUMO (13%)	MLCT/XLCT/L'LCT/IL
			H-5→LUMO (13%)	XLCT/IL/MLCT
$S_0 \rightarrow S_6$	0.087	291.21	H-4→LUMO (98%)	IL/XLCT
$S_0 \rightarrow S_7$	0.020	281.89	HOMO→L+1 (70%)	MLCT/MMCT/ML'CT/L'LCT/L'MCT/IL'/XLCT/XMCT/XL'CT
$S_0 \rightarrow S_{10}$	0.026	263.76	HOMO→L+2 (34%)	MLCT/MMCT/ML'CT/L'LCT/L'MCT/IL'/XLCT/XMCT/XL'CT
			H-2→L+1 (27%)	MLCT/MMCT/ML'CT/L'LCT/L'MCT/IL'
			H-5→LUMO (10%)	XLCT/IL/MLCT
$S_0 \rightarrow S_{11}$	0.034	256.13	H-6→LUMO (77%)	XLCT/IL/MLCT
$S_0 \rightarrow S_{12}$	0.066	252.17	H-2→L+1 (37%)	MLCT/MMCT/ML'CT/ L'LCT/L'MCT/IL'
			HOMO→L+2 (26%)	MLCT/MMCT/ML'CT/L'LCT/L'MCT/IL'/XLCT/XMCT/XL'CT
$S_0 \rightarrow S_{13}$	0.020	247.74	H-4→L+2 (48%)	IL/LMCT/LL'CT/XLCT/XMCT/XL'CT
			H-2→L+2 (15%)	MLCT/MMCT/ML'CT/ L'LCT/L'MCT/IL'
$S_0 \rightarrow S_{14}$	0.040	241.02	H-2→L+2 (23%)	MLCT/MMCT/ML'CT/ L'LCT/L'MCT/IL'

			H-13→LUMO (21%)	IL
			H-7→LUMO (17%)	IL/XLCT
			H-9→LUMO (13%)	IL
$S_0 \rightarrow S_{15}$	0.059	239.79	H-2→L+2 (39%)	MLCT/MMCT/ML′CT/ L′LCT/L′MCT/IL′
			H-13→LUMO (13%)	IL
			H-1→L+2 (13%)	MLCT/MMCT/ML′CT/XLCT/XMCT/XL′CT/L′LCT/L′MCT/IL′/IL/LMCT/LL′CT
$S_0 \rightarrow S_{16}$	0.066	235.90	H-8→LUMO (63%)	XLCT/IL/L′LCT
			H-7→LUMO (12%)	IL/XLCT
$S_0 \rightarrow S_{37}$	0.024	181.56	H-1→L+7 (16%)	MLCT/MMCT/ML′CT/XLCT/XMCT/XL′CT/L′LCT/L′MCT/IL′/IL/LMCT/LL′CT
$S_0 \rightarrow S_{39}$	0.023	179.56	H-4→L+2 (27%)	IL/LMCT/LL′CT/XLCT/XMCT/XL′CT
			H-5→L+2 (11%)	XLCT/XMCT/XL′CT/IL/LMCT/LL′CT/MLCT/MMCT/ML′CT
$S_0 \rightarrow S_{43}$	0.046	177.04	H-15→LUMO (56%)	IL
$S_0 \rightarrow S_{44}$	0.074	176.61	H-3→L+1 (37%)	IL/LMCT/LL′CT

[a] M = Re, L = adcpip, L′ = CO, and X = Cl.

Table S12. DFT-calculated absorptions and character of DFT-calculated transitions for *anti*-[Re]$_2$]; M06-2X/def2TZVP/LANL2DZ for Re/CPCM(THF) level of theory.[a]

**Excited state**	**Oscillator Strength**	**Calculated λ (nm)**	**Transitions (Major Contribution)**	**Assignment**
$S_0 \rightarrow S_3$	0.144	523.08	H-2→LUMO (82%)	MLCT/XLCT/L′LCT
			H-3→LUMO (14%)	MLCT/XLCT/L′LCT/IL
$S_0 \rightarrow S_4$	0.027	512.21	H-3→LUMO (54%)	MLCT/XLCT/L′LCT/IL
			H-4→LUMO (17%)	MLCT/L′LCT
			H-2→LUMO (15%)	MLCT/XLCT/L′LCT
$S_0 \rightarrow S_7$	0.050	372.36	H-6→LUMO (72%)	XLCT/IL
$S_0 \rightarrow S_9$	0.079	329.00	H-7→LUMO (39%)	XLCT/IL
			H-8→LUMO (15%)	XLCT/MLCT/IL
$S_0 \rightarrow S_{10}$	0.022	316.74	H-8→LUMO (74%)	XLCT/MLCT/IL
$S_0 \rightarrow S_{11}$	0.023	302.06	H-9→LUMO (35%)	XLCT/MLCT/IL
			H-11→LUMO (25%)	IL/XLCT/MLCT
			HOMO→L+1 (21%)	MLCT/ML′CT/MMCT/L′LCT/IL′/L′MCT/XLCT/XL′CT/XMCT
$S_0 \rightarrow S_{12}$	0.035	296.10	H-10→LUMO (63%)	XLCT/IL/MLCT/L′LCT
			HOMO→L+1 (12%)	MLCT/ML′CT/MMCT/L′LCT/IL′/L′MCT/XLCT/XL′CT/XMCT
$S_0 \rightarrow S_{14}$	0.038	291.42	HOMO→L+1 (42%)	MLCT/ML′CT/MMCT/L′LCT/IL′/L′MCT/XLCT/XL′CT/XMCT
			H-11→LUMO (25%)	IL/XLCT/MLCT
$S_0 \rightarrow S_{16}$	0.031	280.25	H-12→LUMO (67%)	XLCT/IL/L′LCT/MLCT
$S_0 \rightarrow S_{22}$	0.063	263.05	H-4→L+1 (30%)	MLCT/ML′CT/MMCT/L′LCT/IL′/L′MCT
			H-5→L+1 (19%)	MLCT/ML′CT/MMCT/L′LCT/IL′/L′MCT
$S_0 \rightarrow S_{23}$	0.030	255.89	H-2→L+3 (26%)	MMCT/MLCT/ML′CT/XMCT/XLCT/XL′CT/L′MCT/L′LCT/IL′
			H-3→L+4 (17%)	ML′CT/MMCT/MLCT/XL′CT/XMCT/XLCT/IL′/L′MCT/L′LCT/LL′CT/LMCT/IL
			H-2→L+2 (12%)	MLCT/MMCT/ML′CT/XLCT/XMCT/XL′CT/L′LCT/L′MCT/IL′
$S_0 \rightarrow S_{24}$	0.036	253.70	H-5→L+2 (14%)	MLCT/MMCT/ML′CT/ L′LCT/L′MCT/IL′
			H-1→L+3 (14%)	MMCT/MLCT/ML′CT/XMCT/XLCT/XL′CT/L′MCT/L′LCT/IL′
			H-4→L+2 (12%)	MLCT/MMCT/ML′CT/L′LCT/L′MCT/IL′
$S_0 \rightarrow S_{26}$	0.070	249.04	H-5→L+2 (16%)	MLCT/MMCT/ML′CT/ L′LCT/L′MCT/IL′
			H-3→L+3 (16%)	MMCT/MLCT/ML′CT/XMCT/XLCT/XL′CT/ L′MCT/L′LCT/IL′/LMCT/IL/LL′CT

			H-2→L+4 (14%)	ML′CT/MMCT/MLCT/XL′CT/XMCT/XLCT/IL′/L′MCT/L′LCT/
$S_0 \rightarrow S_{41}$	0.070	205.33	HOMO→L+5 (22%)	MLCT/MMCT/ML′CT/ L′LCT/L′MCT/IL′/XLCT/XMCT/XL′CT
$S_0 \rightarrow S_{42}$	0.027	203.53	H-3→L+1 (19%)	MLCT/ML′CT/MMCT/XLCT/XL′CT/XMCT/L′LCT/IL′/L′MCT/IL/LL′CT/LMCT/
			H-2→L+1 (18%)	MLCT/ML′CT/MMCT/XLCT/XL′CT/XMCT/L′LCT/IL′/L′MCT

[a] M = Re, L = adcpip, L′ = CO, and X = Cl.

Table S13. DFT-calculated absorptions and character of calculated transitions for *syn*-$[Re]_2$; M06-2X/def2TZVP/LANL2DZ for Re/CPCM(THF) level of theory.[a]

**Excited state**	**Oscillator Strength**	**Calculated λ (nm)**	**Transitions (Major Contribution)**	**Assignment**
$S_0 \rightarrow S_3$	0.230	504.09	HOMO→LUMO (95%)	MLCT/IL/XLCT/L′LCT
$S_0 \rightarrow S_7$	0.026	350.80	H-6→LUMO (32%)	IL/XLCT
			H-8→LUMO (19%)	XLCT/IL/MLCT
$S_0 \rightarrow S_9$	0.065	316.50	H-6→LUMO (58%)	IL/XLCT
			H-8→LUMO (29%)	XLCT/IL/MLCT
$S_0 \rightarrow S_{14}$	0.031	285.73	H-10→LUMO (72%)	IL/XLCT/MLCT
			HOMO→L+1 (12%)	MLCT/ML′CT/MMCT/IL/LL′CT/LMCT/XLCT/XL′CT/XMCT/L′LCT/IL′/L′MCT
$S_0 \rightarrow S_{16}$	0.057	282.68	H-8→LUMO (39%)	XLCT/IL/MLCT
			H-14→LUMO (15%)	IL
$S_0 \rightarrow S_{18}$	0.045	268.35	H-4→L+1 (30%)	MLCT/ML′CT/MMCT/IL/L′LCT/IL′/L′MCT
			H-5→L+2 (23%)	MLCT/ML′CT/MMCT/IL/L′LCT/IL′/L′MCT
$S_0 \rightarrow S_{19}$	0.033	267.40	H-4→L+2 (29%)	MLCT/ML′CT/MMCT/IL/L′LCT/IL′/L′MCT
			H-5→L+1 (26%)	MLCT/ML′CT/MMCT/IL/L′LCT/IL′/L′MCT
$S_0 \rightarrow S_{23}$	0.053	255.92	H-2→L+3 (29%)	MLCT/ML′CT/MMCT/XLCT/XL′CT/XMCT/L′LCT/IL′/L′MCT
			H-1→L+4 (17%)	MMCT/MLCT/ML′CT/L′MCT/L′LCT/IL′/XMCT/XLCT/XL′CT
$S_0 \rightarrow S_{24}$	0.040	246.51	HOMO→L+3 (29%)	MLCT/ML′CT/MMCT/IL/LL′CT/LMCT/XLCT/XL′CT/XMCT/L′LCT/IL′/L′MCT
			H-3→L+4 (18%)	MMCT/MLCT/ML′CT/XMCT/XLCT/XL′CT/L′MCT/L′LCT/IL′
			H-1→L+3 (15%)	MLCT/ML′CT/MMCT/L′LCT/IL′/L′MCT/XLCT/XL′CT/XMCT
$S_0 \rightarrow S_{41}$	0.063	205.49	H-3→L+2 (13%)	MLCT/ML′CT/MMCT/XLCT/XL′CT/XMCT/L′LCT/IL′/L′MCT

[a] M = Re, L = adcpip, L′ = CO, and X = Cl.

References

[1] C. Reichardt, Solvatochromic Dyes as Solvent Polarity Indicators, *Chem. Rev.* **1994**, *94*, 2319–2358.
[2] D. M. Manuta, A. J. Lees, Solvatochromism of the Metal to Ligand Charge-Transfer Transitions of Zerovalent Tungsten Carbonyl Complexes. *Inorg. Chem.* **1986**, *25*, 3212–3218.
[3] W. Kaim, S. Kohlmann, S. Ernst, B Olbrich-Deussner, C. Bessenbacher, A Schulz, What determines the solvatochromism of metal-to-ligand charge transfer transitions? A demonstration involving 17 tungsten carbonyl complexes. *J. Organomet. Chem.* **1987**, *321*, 215–226.
[4] U. Mayer, V. Gutmann, W. Gerger, The Acceptor Number – A Quantitative Empirical Parameter for the Electrophilic Properties of Solvents, *Monatsh. Chem.* **1975**, *106*, 1235–1257.
